# The Role of Tumor-Stroma Interactions in Drug Resistance Within Tumor Microenvironment

**DOI:** 10.3389/fcell.2021.637675

**Published:** 2021-05-20

**Authors:** Yanghong Ni, Xiaoting Zhou, Jia Yang, Houhui Shi, Hongyi Li, Xia Zhao, Xuelei Ma

**Affiliations:** ^1^Department of Biotherapy, State Key Laboratory of Biotherapy and Cancer Center, West China Hospital, Collaborative Innovation Center of Biotherapy, Sichuan University, Chengdu, China; ^2^Department of Gynecology and Obstetrics, Development and Related Disease of Women and Children Key Laboratory of Sichuan Province, Key Laboratory of Birth Defects and Related Diseases of Women and Children, Ministry of Education, West China Second Hospital, Sichuan University, Chengdu, China

**Keywords:** tumor microenvironment, antineoplastic drug resistance, cell-cell interplays, CAFs, TAMs, MSCs

## Abstract

Cancer cells resistance to various therapies remains to be a key challenge nowadays. For a long time, scientists focused on tumor cells themselves for the mechanisms of acquired drug resistance. However, recent evidence showed that tumor microenvironment (TME) is essential for regulating immune escape, drug resistance, progression and metastasis of malignant cells. Reciprocal interactions between cancer cells and non-malignant cells within this milieu often reshape the TME and promote drug resistance. Therefore, advanced knowledge about these sophisticated interactions is significant for the design of effective therapeutic approaches. In this review, we highlight cancer-associated fibroblasts (CAFs), tumor-associated macrophages (TAMs), tumor-associated neutrophils (TANs), myeloid-derived suppressor cells (MDSCs), T-regulatory lymphocytes (Tregs), mesenchymal stem cells (MSCs), cancer-associated adipocytes (CAAs), and tumor endothelial cells (TECs) existing in TME, as well as their multiple cross-talk with tumor cells, which eventually endows tumor cells with therapeutic resistance.

## Introduction

Over decades, cancer has always been a major public health problem worldwide. According to the statistics reported by the American Cancer Society in 2020, there are nearly 1.8 million new cancer cases and 600 thousand cancer deaths in the United States (Siegel et al., 2020). In general, antitumor treatments mainly include surgery, chemotherapy, radiotherapy, immunotherapy, hormone therapy and targeted therapy. Patients can obtain more obvious effects in the initial stage of treatment, while the emergence of drug resistance often becomes an inevitable obstacle to clinical recovery in the late stage. For a long time, scientists focused on tumor cells themselves for the mechanisms of acquired drug resistance, such as the up-regulation of drug efflux pump protein (Amawi et al., 2019), epigenetic abnormalities (Shah and Rawal, 2019), oncogenic mutations and tumor heterogeneity etc. (Hientz et al., 2017; Zhang H. et al., 2017; Vaidya et al., 2020). The concept of tumor microenvironment (TME) was first proposed by Stephen Paget who indicated that the relationship between breast cancer and its microenvironment is like “seed and soil” (Paget, 1989). TME refers to the local biological environment where tumor cells exist and the number of cancer cells varies from 5 to 100% (Ambrose et al., 2015). It is currently considered to be an acidic and hypoxic milieu infiltrated by cancer cells and accessory cells, including stromal cells, endothelial cells, immune cells, mesenchymal cells, adipocytes and pericytes, as well as extracellular matrix and cytokines (Hui and Chen, 2015; Yamaguchi and Perkins, 2018).

**FIGURE 1:**
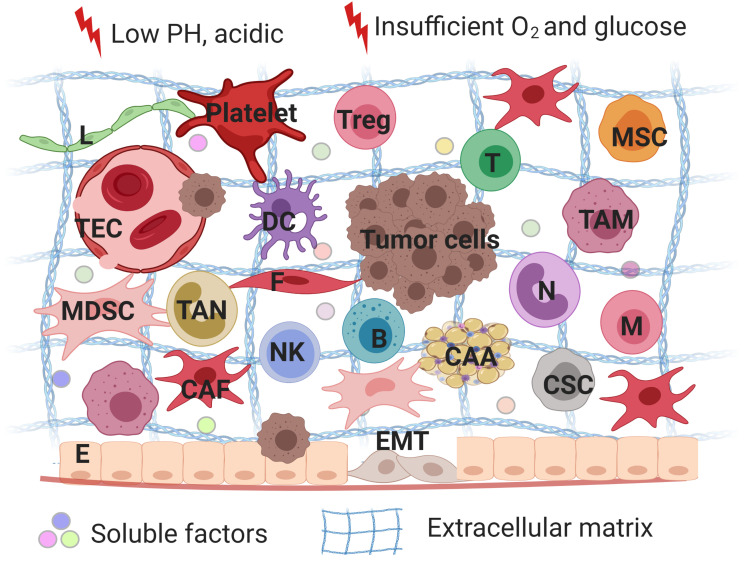
A schematic overview of tumor microenvironments. Tumor microenvironment refers to the local biological environment where tumor cells exist, and are composed of tumor cells, stromal cells and immune cells. TME is involved in the initiation and maintenance of non-cell autonomous resistance by a variety of mechanisms, including hypoxia, low PH, shifts and polarizations in the immune cell population, vascular abnormalities and diverse stroma cells-derived secretomes, exosomes, soluble factors. TME serves as safeguard to tumor cells by providing mechanical support or secreting different cytokines to evade treatment. Treg, regulatory T lymphocytes; MSC, mesenchymal stem cells; T, T lymphocytes; B, B lymphocytes; DC, dendritic cells; N, neutrophils; M, macrophages; NK, natural killer cells; E, epithelial cells; F, fibroblasts; CAA, cancer-associated adipocytes; L, lymphatic endothelial cells; TEC, tumor endothelial cells; CSC, cancer stem cells; TAM, tumor-associated macrophages; CAF, cancer-associated fibroblasts; MDSC, myeloid-derived suppressor cells; EMT, epithelial-mesenchymal transition. The figure was created with BioRender.com.

Tumor microenvironment serves as safeguard to tumor cells by providing mechanical support or secreting different cytokines to evade treatment (Figure 1; Kodet et al., 2018). Diverse heterogeneous cells along with their secretory cytokines constitute a complex network, which allows tumor cells to proliferate rapidly, maintain stemness, insensitive to medication and escape from immune surveillance. In fact, non-inherent-adaptive resistance, also called non-cell-autonomous resistance, is therapeutically dependent (Calcinotto et al., 2018). TME is involved in the initiation and maintenance of non-cell autonomous resistance by a variety of mechanisms, including hypoxia, low PH, shifts and polarizations in the immune cell population, vascular abnormalities and diverse stroma cells-derived secretomes, exosomes, soluble factors. In order to deeply understand progression and drug resistance of tumor cells, a thorough knowledge of the interactions between tumor cells and their microenvironment is needed. Therefore, we reviewed a series of non-malignant cells constituting the tumor microenvironment and how they interplay with tumor cells to promote therapeutic resistance with an expectance to spark new ideas of designing more specific approaches for cancer.

## Cancer Associated Fibroblasts (CAFs) and Drug Resistance

Cancer associated fibroblasts (CAFs) are one of the most important components of TME. Numerous researches have suggested its role in forming the communication networks with tumor cells and other elements in TME, which is responsible for therapeutic resistance in pancreatic cancer, colorectal neoplasm, breast cancer, ovarian neoplasm, lung cancer, gastric cancer and so on (Mao et al., 2015; Zhou B. et al., 2016; Wen et al., 2017; Cazet et al., 2018; Tang et al., 2018; Wei et al., 2018; Yi et al., 2018; Hu et al., 2019; Wang L. et al., 2019). The origins of CAFs are diverse. In addition to the normal activated fibroblasts, marked by the expression of α-smooth muscle actin (α-SMA), CAFs can also evolve from other cell types, such as bone marrow-derived cells (BMDCs) (Mishra et al., 2008), epithelial cells undergone epithelial-mesenchymal transition (EMT) (Kalluri and Neilson, 2003), endothelial cells following endothelial-to-mesenchymal transition (EndMT) (Zeisberg et al., 2007), adipocytes and stellate cells (Yin et al., 2013). As a result, the subgroups of CAFs are highly heterogenous which makes it difficult to identify a specific population of CAFs in TME (Fiori et al., 2019). Currently recognized biological hallmarks of CAFs include α-SMA, fibroblast-specific protein 1 (FSP1/S100A4), platelet-derived growth factor receptor (PDGFR) and type-I collagen etc. (Sugimoto et al., 2006).

Briefly, the intricate mechanisms of antineoplastic drug resistance mediated by CAFs can be summarized into the following aspects: (1) secreting soluble factors; (2) remodeling extracellular matrix (ECM); (3) reprogramming the metabolic process of tumor cells; (4) inducting epigenetic modifications of tumor cells; (5) delivering exosomes.

### Secreting Soluble Factors

**FIGURE 2:**
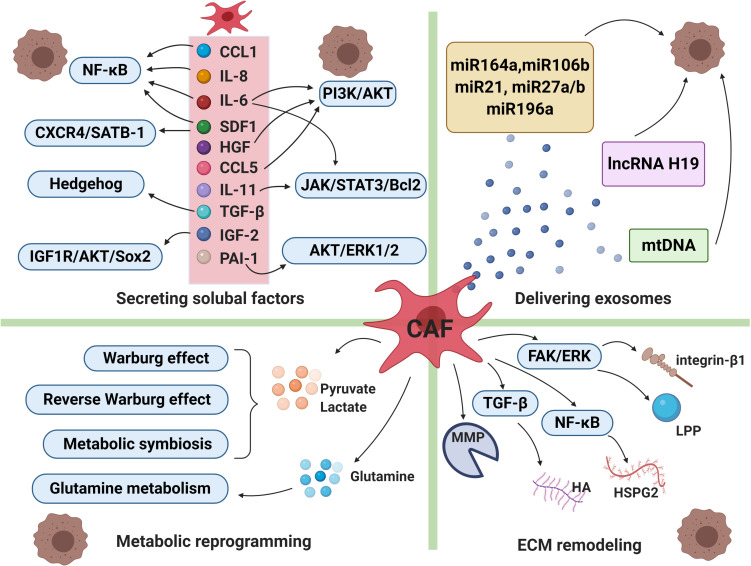
Cancer-associated fibroblasts and drug resistance. The intricate mechanisms of drug resistance mediated by CAFs include secreting soluble factors, delivering exosomes, metabolic reprogramming and extracellular matrix (ECM) remodeling. CAFs can secret a broad range of cytokines or factors which enable to activate a series of signaling cascades, leading to therapeutic resistance. CAFs-derived exosomes deliver miRNAs, lncRNAs and mtDNA to cancer cells, which participate in transmitting paracrine signals of therapeutic resistance. Moreover, in order to adapt to a glucose-deficient microenvironment, CAFs coordinate with tumor cells to modulate metabolic mode. Lastly, activated signals within CAFs increased production of extracellular matrix components, resulting in changes of its physical and biochemical characteristics under therapeutic pressure. CCL1, chemokine C-C motif ligand-1; CCL5, chemokine C-C motif ligand-5; IL-6, interleukin-6; IL-8, interleukin-8;IL-11, interleukin-11; SDF1, stromal cell derived factor-1; HGF, hepatocyte growth factor; TGF-ß, transforming growth factor-β; IGF-2, insulin-like growth factor 2; PAI-1, plasminogen activator inhibitor-1, MMP, matrix metalloproteinase; HA, hyaluronan; HSPG2, heparin sulphate proteoglycan 2; LPP, lipoma-preferred partner. The figure was created with BioRender.com.

It has been proven that CAFs can secret various cytokines or factors which enable to activate a series of signaling cascades, leading to drug resistance and tumor cells relapse eventually. CAFs secrete diverse growth factors (including fibroblast growth factor, hepatocyte growth factor, transforming growth factor, and stromal cell derived factor), cytokines (such as IL-6, IL-8, IL-10 etc.), chemokines and protease (Figure 2; Mathot et al., 2017).

IL-6 was described to induce drug resistance. Multiple pathways such as nuclear factor-κB (NF-κB), JAK/STAT3, PI3K/AKT have been activated under therapeutic pressure in breast cancer and non-small cell lung cancer (Mao et al., 2015; Shien et al., 2017). Secretion of IL-6 causes the upregulation of C-X-C motif chemokine receptor 7 (CXCR7) through STAT3/NF-κB pathway. The expression of CXCR7 in esophageal squamous cell carcinoma tissues from chemotherapy resistant patients was significantly higher than that of chemotherapy sensitive patients (Qiao et al., 2018). In addition, the potential of IL-6 to drive epithelial-mesenchymal transition (EMT) has been identified in esophageal adenocarcinoma (Ebbing et al., 2019). IL-8 is another cytokine relating to CAFs. High levels of IL-8 were detected in gastric cancer patients who were insensitive to platinum-based therapy, which was associated with NF-κB activation and increased expression of ATP-binding cassette subfamily B member 1 (ABCB1) (Zhai et al., 2019). Moreover, in gastric cancer and lung adenocarcinoma, CAFs drive chemoresistance through IL-11 secretion via IL-11/STAT3/Bcl2 signaling pathway (Tao et al., 2016; Ma et al., 2019).

Stromal cell derived factor-1 (SDF-1) was found to be overexpressed in CAFs and played an essential role in tumor cell metastasis and chemoresistance by activating its receptor chemokine receptor 4 (CXCR4) (Orimo and Weinberg, 2006; Teng et al., 2016; Zou et al., 2017). The expression level of SDF-1 in CAFs was regulated by microRNA mir-1 negatively, with NF-κB and Bcl-xL signaling pathways involved in lung cancer chemoresistance (Li et al., 2016). Wei et al. found that SDF-1 secreted by CAFs promoted chemoresistance through paracrine induction of special AT-rich sequence-binding protein 1 (SATB-1) in pancreatic tumor cells. In turn, SDF-1/CXCR4/SATB-1 axis helped to maintain CAFs characteristics. Therefore, a reciprocal feedback loop was formed (Wei et al., 2018).

Transforming growth factor-β (TGF-β) expression in cancers is generally regarded as an important therapeutic target because it plays a key role in regulating the proliferation, differentiation and survival of cancer cells (Ciardiello et al., 2020; Li et al., 2020). CAFs conferred esophageal squamous cell carcinoma cells significant chemoresistance by activating FOXO 1, a member of the forkhead transcription factors in the O-box sub-family, inducing the secretion of TGF-β1 (Zhang H. et al., 2017). Hypoxia-inducible factor (HIF-1α) has been shown to synergize with CAFs paracrine signals, namely TGF-β2, to activate hedgehog transcription factor GLI2 in cancer stem cells (CSCs), leading to enhanced stemness/dedifferentiation and intrinsic resistance to chemotherapy of colorectal cancer (Tang et al., 2018). Apart from their assembly effect, CAFs also play a part in HIF-1 pathway modulation which mediates the photodynamic treatment resistance of colorectal cancer (Lamberti et al., 2019).

Hepatocyte growth factor (HGF) is one of the growth factors derived from CAFs. Recent evidence has revealed the role of HGF in transmitting resistance to epidermal growth factor receptor (EGFR) targeted therapy in lung cancer (Kasahara et al., 2010; Yi et al., 2018). HGF was identified to be a major mediator of CAFs induced resistance to EGFR tyrosine kinase inhibitor (EGFR-TKI) (Apicella et al., 2018; Suzuki et al., 2019). HGF derived from CAFs activated the c-Met/PI3K/Akt and glucose-regulated protein 78 (GRP78) pathways of SKOV3 and HO-8910 cells to attenuate the inhibition of cell proliferation and apoptosis caused by paclitaxel (PAC). Furthermore, HGF induced drug resistance has been validated in nude mice (Deying et al., 2017).

Cancer-associated fibroblasts have been found to constrict cisplatin-induced ovarian cancer cell apoptosis through activating STAT3 signaling (Yan et al., 2016). Consistently, another study demonstrated that cisplatin resistance in ovarian cells was attributed to the secretion of chemokine C-C motif ligand-5 (CCL5) by CAFs. Its molecular mechanism related to enhanced STAT3 and PI3K/Akt phosphorylation levels in ovarian cells (Zhou B. et al., 2016). Chemokine C-C motif ligand-1 (CCL1), combining exclusively with chemokine (C-C motif) receptor 8 (CCR8), is an inflammatory factor. However, its role has already gone beyond inflammation (Korbecki et al., 2020). Li et al. (2018) suggested that CCL1 participated in CAFs mediated chemoresistance by activation of TGF-β/NF-κB signaling pathway in colorectal cancer.

Besides the factors mentioned above, CAFs are capable of secreting many other humoral elements to induce therapeutic resistance. Evidence of increased expression of insulin-like growth factor 2 (IGF2) and insulin-like growth factor receptor-1 (IGF-1R) in CAF accumulating-lung cancer tissues proved that CAFs promote chemoresistance through IGF2/IGF-1R signal. It led to the activation of AKT/SoX2 pathway, elevating the expression of P-glycoprotein, decreasing drug retention and increasing drug efflux ultimately (Zhang Q. et al., 2018). Midkine (MK) is a kind of heparin-binding growth factor, promoting carcinogenesis and chemoresistance. CAFs in TME lead to high levels of MK in tumors. As a result, CAF-derived MK can induce cisplatin resistance through increased expression of lncRNA (Zhang D. et al., 2017). It has also shown that cisplatin treatment triggered the AKT and ERK1/2 signal cascades in esophageal squamous cell carcinoma cells mediated by releasing of plasminogen activator inhibitor-1 (PAI-1) from CAFs. The study confirmed that there is relevance between elevated PAI-1 level in CAFs and poor progression-free survival after therapy (Che et al., 2018). When cultured head and neck squamous cell carcinoma (HNSCC) cell lines with CAFs, cetuximab resistance was induced and elevated level of matrix metalloproteinase-1 (MMP-1) was detected, and the resistance can be reversed by using MMP inhibitor. The result suggested that drug resistance was achieved by CAFs derived MMP family (Johansson et al., 2012).

### Remodeling Extracellular Matrix

Extracellular matrix is a dynamic system which changes its physical and biochemical characteristics under pathological conditions, for example, its molecular composition and elasticity (Gilbert et al., 2010). ECM can also sufficiently impede drug delivery by increasing microvascular endothelial cell remodeling (Figure 2). A study on ovarian cancer chemoresistance showed that CAFs increased the expression of lipoma-preferred partner (LPP) in endothelial cells through microfibrillar associated protein 5 (MFAP5)/FAK/ERK/LPP signaling pathway. Mechanically, LPP enhanced the formation of focal adhesion and stress fibers, promoting the mobility and permeability of endothelial cells. As a result, paclitaxel delivery to malignant cells has been decreased via increased intra-tumoral microvascular leakiness (Leung et al., 2018). In addition, hyaluronan (HA) has also been found to participate in remodeling extracellular matrix by producing high interstitial fluid pressure (IFP) to reduce the amount of drug exchange through capillaries (Paunescu et al., 2011; Jacobetz et al., 2013). CAFs are involved in producing HA in a TGF-β dependent manner (Sapudom et al., 2020). Meanwhile, CAFs are found to delay cancer cell response to chemotherapy through another critical component in matrix, heparin sulfate proteoglycan 2 (HSPG2). Stromal deposition of HSPG2 was partly mediated by NF-κB signaling, which enhanced the invasion, metastasis of pancreatic cells and induced gemcitabine resistance (Vennin et al., 2019). CAFs in BRAF mutant melanoma may participate in resistance to BRAF inhibitor PLX4720 by generating fibronectin-rich matrix. Melanoma-associated fibroblasts conferred PLX4720 tolerance dependent on the upregulation of integrin-β1 and the activation of focal adhesion kinase (FAK)/Src/ERK signaling pathway (Hirata et al., 2015).

### Reprogramming the Metabolic Process of Tumor Cells

The abnormal proliferation of tumor cells relies on the enhanced adaptation to the nutritional microenvironment mediated through reprogrammed metabolic process. As a result, alterations in energy metabolism are considered to be one of the characteristics of tumor cells (Hanahan and Weinberg, 2011). In order to adapt to a glucose-deficient microenvironment, cancer cells undergo a metabolic switch to aerobic glycolysis, also known as the Warburg effect (Siska et al., 2020). Lactate is the end product of aerobic glycolysis and increased lactate production in MET/EGFR TKI-resistant breast cancer cells has been observed. Interestingly, lactate served as a critical media between breast cells and CAFs. Specifically, lactate uptake by CAFs activates NF-κB signals of CAFs, resulting in the upregulation of HGF. In turn, increased HGF turns on MET signals in cancers and confers resistance to TKIs (Apicella et al., 2018).

Although Warburg effect is a distinctive character of tumor cells, recent studies showed that other metabolic features, notably the reverse Warburg effect, metabolic symbiosis, and glutamine metabolism, pose challenges to antitumor therapy due to adaptive or acquired drug resistance (Figure 2; Yoshida, 2015). Migneco proposed a new model for understanding the reverse Warburg effect, in which glycolytic CAFs secreted pyruvate/lactate used by adjacent epithelial cancer cells as a source of mitochondrial tricarboxylic acid cycle (TCA) cycle, oxidative phosphorylation and ATP production. The result suggested that metabolic products produced by glycolytic CAFs promote tumor cells to escape from antiangiogenic drugs via decreasing the reliance of cancer cells on a vascular blood supply (Migneco et al., 2010). Metabolic symbiosis of CAFs with epithelial cancer cells has been identified in (monocarboxylate transporter 1) MCT1-positive prostate tumor cells and MCT4-positive CAFs (Pértega-Gomes et al., 2014). MCT4-positive hypoxic cells create a low PH acidic microenvironment through aerobic glycolysis and secreting lactate, while MCT1-positive oxidative cells take up lactate as a substrate for TCA cycle, which is termed as metabolic symbiosis (Montenegro and Indraccolo, 2020). Besides, it has been proved that angiogenesis inhibitors resistance mediated by metabolic symbiosis was dependent on mTOR signaling (Allen et al., 2016; Jiménez-Valerio et al., 2016). Tumor cells also achieve metabolic process via glutamine metabolic reprogramming. For example, in prostate cancer, the increase in glutamine synthesis after macropinocytosis of extracellular fluid was detected in CAFs, which was related to the cascade activation of RAS signaling. In turn, CAFs-secreting glutamine promoted mitochondrial metabolism in prostate cancer cells, inducing neuroendocrine differentiation, facilitating the development of resistance to androgen deprivation therapy (ADT). In consistence, prostate cancer patients with ADT resistance displayed increased blood glutamine level than those with satisfactory treatment outcomes (Mishra et al., 2018).

### Inducting Epigenetic Modifications of Tumor Cells

Accumulating evidence underscores the epigenetic modification of tumor cells induced by CAFs plays a part in drug resistance. Dysregulated DNA methylation in CAFs has been reported to be concerned with breast cancer, stomach cancer and pancreatic cancer (Liu M. et al., 2017). It is proved that DNA methylation level in stromal cells is related to that of adjacent cancer cells (Hanson et al., 2006; Rodriguez-Canales et al., 2007). Soluble factors secreted by CAFs obviously upregulated the level of methylated CpGs on their regulated gene region (372 genes) in breast cancer cells, which shed light on the importance of epigenetic modifications of tumor cells induced by CAFs (Mathot et al., 2017). In addition, factors derived from CAFs were capable to induce reversible DNA hyper- and hypo-methylation during (epithelial-to-mesenchymal transition) EMT and (mesenchymal-to-epithelial transition) MET, leading to castration resistance and cancer stem cells expansion, predicting poor prognosis in prostate cancer (Pistore et al., 2017). Much evidence has already proven that EMT and stemness are also major contributors to drug resistance (Shibue and Weinberg, 2017; Nilsson et al., 2020). Moreover, EMT process was mediated by TGF-β derived from CAFs. Extensive methylation changes following TGF-β releasing were detected in ovarian cancer cells. Treatment with DNA methyltransferases inhibitor blockades DNA methylation in the EMT process (Cardenas et al., 2014). To develop a better therapeutic approach, a deeper knowledge of the impact of epigenetic regulation on the tumor microenvironment is necessary for combining epigenetic and checkpoint blockage therapies.

### Delivering Exosomes

Cancer-associated fibroblasts derived exosomes have also emerged as active mediators of cancer progression and drug resistance by transmitting paracrine signals. Exosomes are membrane vesicles with a diameter of approximately 30-100 nm, containing protein, DNA, mRNA and miRNA (Fu et al., 2016; Kalluri, 2016; Li and Nabet, 2019). Exosomes are taken up by adjacent cells through endocytosis and act on the recipient cells. Recent studies have proven that exosomes are involved in the dynamic interplays between CAFs and cancer cells to promote tumor progression and chemoresistance (Figure 2; Ren et al., 2018).

Dysregulation of miRNAs in CAFs and the transfer of exosomal miRNAs between cancer cells and the microenvironment are associated with chemoresistance in multiple cancer types (Tanaka et al., 2015; Zhang L. et al., 2018; Qin et al., 2019; Guo et al., 2020). In pancreatic cancer, gemcitabine (GEM) treatment induced resistant CAFs that released exosomes containing SNAI1 and miRNA 164a. SNAI1 expression in exosomes was significant in inducing EMT. Moreover, the resistance can be reversed by treatment with GW4869, an inhibitor of exosome release (Richards et al., 2017; You et al., 2019). miRNA 106b was delivered directly from CAFs to pancreatic tumor cells via exosomes, leading to GEM insensitivity of tumor cells by targeting TP53INP1 (Fang et al., 2019). Except for miRNA 164a and miRNA 106b, miRNA 21 was also reported in GEM-induced chemoresistance of pancreatic cancer (Zhang L. et al., 2018). In ovarian neoplasm, miRNA 21 derived from CAFs and cancer associated adipocytes was transferred to cancer cells, which conferred paclitaxel resistance by directly targeting apoptotic protease activating factor-1(APAF1) (Yeung et al., 2016). Further, it has been demonstrated that cisplatin and paclitaxel treatment of gastric cancer promoted the secretion of miRNA 522 from CAFs, with the activation of ubiquitin-specific protease 7 (USP7)/heterogeneous nuclear ribonucleoprotein A1 (hnRNPA1) axis, resulting in arachidonate lipoxygenase 15 (ALOX15) inhibition and ultimately decreased chemo-sensitivity (Zhang H. et al., 2020). Additionally, miRNA 27a/b over-expressed CAFs in esophageal cancer microenvironment were correlated with cisplatin resistance by upregulation of TGF-β (Tanaka et al., 2015). In head and neck cancer, cisplatin resistance was found to be linked exosomal miRNA 196a and its targets, CDKN1B and ING5 (Qin et al., 2019).

Long non-coding RNA signatures of tumor-derived exosomes have been identified to be responsible for drug resistance. Exosome-enriched lncRNA H19 and colorectal-associated lncRNA can be transferred from CAFs to adjacent colorectal cells, inducing stemness of CSC and chemoresistance by activating Wnt/ß-catenin signaling pathway (Ren et al., 2018; Deng et al., 2020). Besides, mitochondrial DNA (mtDNA) transfer from CAFs to hormonal therapy-resistant breast cancer cells occurred via exosomes. Specifically, cancer cells with impaired metabolism received the exosomes loaded with full mitochondrial genome, causing restoration of oxidative phosphorylation. The delivery of mtDNA promoted resistance to hormone therapy and induced self-renewal potential of breast cancer cells (Sansone et al., 2017).

In short, exosomes transfer and secretion of soluble molecules constitute a major communication channel between CAFs and tumor cells as well as other stromal components in TME, which promotes cancer progression and therapeutic resistance.

Based on these multiple effects of CAFs on drug resistance (Table 1), CAF have become an interesting therapeutic target for cancer intervention. Here, we will briefly discuss the main advances in CAF-targeted therapeutic strategies. Firstly, direct elimination of CAFs can significantly enhance the efficacy of chemotherapy. Co-delivery of cyclopamine (CPA), a kind of sonic hedgehog inhibitor, and paclitaxel (PTX) by construction of polymeric micelle (M-CPA/PTX) shows promising therapeutic outcomes for Pancreatic ductal adenocarcinoma (PDAC). M-CPA can deplete CAFs and modulate stroma thus to enhance the cytotoxic activity of PTX. *In vivo*, the compounds remarkably prolonged the survival time and inhibited tumor growth (Zhao et al., 2018). However, in a hybrid mouse model of spontaneous PDAC mice with α-SMA thymidine kinase (TK) transgenic mice, depletion of myofibroblasts reduced the production of type I collagen and altered ECM, with enhanced tumor hypoxia, immunosuppression and reduced survival (Özdemir et al., 2015). From this point of view, it seems that we need to be more cautious about the strategy of exhausting CAFs. Secondly, given the potential risks of depleting CAFs, normalization or inactivation of activated CAFs is another choice for regulating the functions of CAFs. By loading fibroblast activation protein-α (FAP-α) antibody and CXCL12 siRNA onto the peptide nanoparticles (PNP), the nanosystem (PNP/siCXCL12/mAb) can selectively downregulate the expression of CXCL12 in CAFs via FAP-α recognition, causing inactivation of CAFs and blockade of CAFs-related tumor metastasis. As such, attempts to inactivate or normalize CAFs rather than diminish them, may be more preferable approaches and can promote the development of novel anticancer therapy. Thirdly, it is feasible to restore chemotherapy sensitivity of tumor cells by blockading the cross-talk between CAFs and tumor cells. IL-1β and TGF-β1 pathways are involved in CAFs recruitment and polarization. Simultaneous inhibition of IL-1β and TGF-β1 pathways with TAK1 inhibitor plus TGFBR1 inhibitor could restrain the activation of NF-κB, reduce the secretion of IL-6 and IL-11, and sensitize colorectal cancer tumor cell line (DLD1) to oxaliplatin. *In vivo*, the combined therapy diminished colorectal tumor cells metastasis and CAFs aggregation (Guillén Díaz-Maroto et al., 2019).

**TABLE 1:** Mechanisms of antitumor drug resistance mediated by CAFs.

Tumor type	Drug resistance	Mechanisms	References
Breast cancer	Trastuzumab	CAFs secreting IL-6, thus activating NF-κB, JAK/STAT3 and PI3K/AKT pathways.	Mao et al., 2015
	Hormonal therapy	mtDNA transfer from CAFs to cancer cells via exosomes.	Sansone et al., 2017
CRC	Photodynamic therapy	CAFs reducing the sensitivity of cancer cells to photodynamic activity.	Lamberti et al., 2019
	Oxaliplatin	CAFs expressing lncRNA, activating Wnt/β-catenin pathway.	Deng et al., 2020
ESCC	Cisplatin	CAFs secreting IL-6, upregulating CXCR7 through STAT3/NF-κB pathway.	Qiao et al., 2018
	Cisplatin	CAFs secreting PAI-1 activating AKT and ERK1/2 signal.	Che et al., 2018
Gastric cancer	Cisplatin	CAFs secreting IL-8, activating NF-kB signal and increasing the expression of ABCB1.	Zhai et al., 2019
	Chemoresistance	CAFs expressing IL-11 and activating IL-11/IL-11R/gp130/JAK/STAT3 signal.	Ma et al., 2019
	Cisplatin, paclitaxel	CAFs-derived miRNA 522 leading to ALOX15 suppression and decreased lipid-ROS accumulation.	Zhang H. et al., 2020
Lung adenocarcinoma	EGFR-TKI	CAFs expressing HGF.	Suzuki et al., 2019
	Cisplatin	CAFs expressing IL-11 and activating IL-11/IL-11Rα/STAT3 signal.	Tao et al., 2016
Melanoma	PLX4720	CAFs upregulating integrin-β1, activating FAK/Src/ERK pathway.	Hirata et al., 2015
NSCLC	Selumetinib, erlotinib	CAFs secreting IL-6 inducing EMT switch via JAK1/STAT3 activation.	Shien et al., 2017
	Crizotinib, erlotinib	CAFs utilizing lactate to increase HGF expression.	Apicella et al., 2018
Ovarian cancer	Paclitaxel	miRNA 21 derived from CAFs binding directly to its novel target APAF1.	Au Yeung et al., 2016
	Paclitaxel	CAF-derived HGF activating c-Met/PI3K/Akt and GRP78 signal.	Deying et al., 2017
PDAC	Gemcitabine	CAFs-derived exosomes containing Snail and microRNA-146a, inducing EMT.	Richards et al., 2017
	Gemcitabine	CAFs increasing the expression of HSPG2 via NF-κB signal.	Vennin et al., 2019
	Gemcitabine	CAFs-derived SDF-1 promoting cancer cells SATB-1 expression.	Wei et al., 2018
Prostate cancer	Castration resistance	CAFs inducing DNA hyper- and hypo-methylation during EMT and MET.	Pistore et al., 2017

*CRC, colorectal cancer; ESCC, esophageal squamous cell carcinoma; PAI-1, plasminogen activator inhibitor-1; CXCR7, C-X-C motif chemokine receptor 7; ABCB1, ATP-binding cassette subfamily B member 1; ALOX15, arachidonate lipoxygenase 15; EGFR-TKI, epidermal growth factor receptor tyrosine kinase inhibitor; NSCLC, non–small cell lung cancer; EMT, epithelial-to-mesenchymal transition; HGF, hepatocyte growth factor; APAF1, apoptotic protease activating factor-1; GRP78, glucose-regulated protein 78; PDAC, pancreatic ductal adenocarcinomas; HSPG2, heparin sulfate proteoglycan 2; SDF-1, stromal cell derived factor-1; SATB-1, special AT-rich sequence-binding protein 1.*

## Immune Inflammatory Cells and Drug Resistance

### Tumor Associated Macrophages (TAMs)

Tumor associated macrophages are the most abundant immune cells found in TME (Perrotta et al., 2018; Najafi et al., 2019). Increased TAMs infiltration within TME is declared to be concerned with poor prognosis and unsatisfactory response to chemotherapies for cancer patients (Sugimura et al., 2015; Li et al., 2019). TAMs derive from the circulating Ly6C^+^CCR2^+^ monocytes. TAMs are characterized with great heterogeneity in TME and they can be separated into two subgroups conventionally: M1 and M2 (Liu and Cao, 2015). M1-type macrophages are activated via classical pathway while M2-type macrophages are alternatively activated. In general, the role of M2 phenotype is defined to promote tumor progression and drug resistance, to produce anti-inflammatory cytokines, and to induce Th2 response. Macrophage polarization from M1 to M2 state is common in cancer condition educated by microenvironment (Moradi-Chaleshtori et al., 2020).

The interaction between tumor cells and TAMs which associated with therapeutic resistance lies on that tumor cells promote TAMs to differentiate into immunosuppressive M2-polarized macrophages under treatment pressure. In turn, M2 TAMs endow cancer cells with acquired chemoresistance through diverse mechanisms. IL-34 secreted by chemo-resistant tumor cells in a paracrine manner enhanced the polarization of M2 TAMs by activating colony stimulating factor 1 receptor (CSF1R)/AKT signaling pathway (Baghdadi et al., 2016). Besides, elevated expression of macrophage inhibitory factor (MIF) was detected in cisplatin resistant lung cancer cells, which mediated M2 polarization of TAMs through Src/CD155/MIF signaling (Huang et al., 2019). In aromatase inhibitors resistant breast cancer cells, Jagged1-Notch pathway was activated, leading to macrophage differentiation toward M2 TAMs and upregulated expression of IL-10. M2 TAMs, in turn, contributed to the acquisition of drug resistance and metastasis (Liu H. et al., 2017). In gastric cancer treated with 5-fluorouracil (5-FU), transformation of M2-type TAMs was ascribed to accumulation of reactive oxygen species (ROS), activating hypoxia-inducible factor 1α (HIF-1α) signaling, driving the expression of high-mobility group box 1(HMGB1). As a result, tumor-protected M2-type TAMs produced growth differentiation factor 15 (GDF15) to enhance the fatty acid β-oxidation in tumor cells, which induced chemoresistance (Yu et al., 2020).

Like CAFs, TAMs secret numerous soluble factors into TME to protect tumor cells from drug attack, including enzymes, exosomes, interleukins, chemokines etc. It was found that cathepsin B and S expressing macrophages protected against paclitaxel-induced tumor cells death. The combined application of paclitaxel and cathepsin inhibitor can effectively enhance the therapeutic effect (Shree et al., 2011). Halbrook et al. (2019) pointed that alternatively activated TAMs secreting a series of pyrimidine nucleosides conferred pancreatic tumor cells resistance to gemcitabine, through molecular competition at the level of drug uptake and metabolism. TAMs also induced upregulation of cytidine deaminase, mediating inactivation of gemcitabine (Weizman et al., 2014). In colorectal cancer cells, 5-fluorouracil (5-FU) promotes the production of putrescine by TAMs. As a result, putrescine endows resistance to 5-FU by inhibiting the activation of p-JNK/caspase-3 apoptosis pathway (Zhang et al., 2016). M2-polarized TAMs are found to produce NO to protect tumor cells from apoptosis induced by cisplatin. The mechanism lies on NO/cGMP/PKG signaling pathway, leading to the inhibition of acid sphingomyelinase (Perrotta et al., 2018). Exosomal transfer of miRNA from macrophages to tumor cells has been noted as well. TAMs-derived exosomes transfer miR-365 to pancreatic adenocarcinoma cells to elicit gemcitabine resistance, which is performed by enhanced expression of triphosphate nucleotide and cytidine deaminase (Binenbaum et al., 2018). In addition, exosomes containing miR-223 and miR-21 are demonstrated to be associated with PTEN-PI3K/AKT signaling pathway in cisplatin chemoresistance of epithelial ovarian cancer and gastric cancer cell, respectively (Zheng et al., 2017; Zhu et al., 2019). Macrophages are usually recruited into TME and secret various chronic inflammatory cytokines, both pro-inflammatory and anti-inflammatory factors. Angst et al. (2008) highlighted that TAMs protect pancreatic cancer cells from apoptosis by secreting IL-1ß, a pro-inflammatory cytokine, increasing the expression of cyclooxygenase-2 (COX-2) and prostaglandin E2 (PGE2) through activating MAPK and ERK1/2 pathway. It has been observed that the dysregulation of miR155-5p/C/EBPb/IL-6 signaling in TAMs was responsible for chemoresistance in colorectal cancer. As a result, TAMs secreted IL-6 to activate IL-6R/STAT3/miR-204-5p signaling pathway in tumor cells (Yin et al., 2017). Hedgehog pathway inhibitor cyclopamine treatment increased the infiltration of M2-type TAMs in breast cancer, attenuating the efficacy of chemotherapy due to the accumulation of IL-6 (Xu X. et al., 2019). Besides, IL-10/STAT3/bcl-2 signaling pathway has been proven to be linked with breast cancer cell resistance to paclitaxel. Inhibition of TAMs derived-IL-10 using neutralizing antibody significantly enhanced sensitivity of breast cancer cells (Yang et al., 2015). The cross-talk between TAMs and HNSCC cells was mediated by IL-6 and C-C motif chemokine ligand 15 (CCL15). HNSCC cells secret (vascular endothelial growth factors) VEGF to recruit macrophages and induce their polarization by secreting IL-6. Polarized TAMs produce CCL15 to weaken the sensitivity of tumor cells to gefitinib via CCL15/CCR1/NF-κB signaling pathway (Yin et al., 2019).

Targeting of immune-regulatory receptors, for example, programmed cell death protein-1 (PD-1) and its ligand PD-L1, as well as cytotoxic T-lymphocyte-associated antigen-4 (CTLA-4), reduces T cell suppression, and relives T cell function, thereby restoring antitumor immunity. However, accumulating reports have displayed that TAMs may account for drug resistance to immune checkpoint blockades (ICBs). Wu et al. demonstrated HIF-1α enhanced the level of triggering receptor expressed on myeloid cells-1 (TREM-1) of TAMs. On the one hand, TREM-1^+^ TAMs damaged the cytotoxic functions of CD8^+^ T cells and caused CD8^+^ T cell apoptosis, in a PD-1/PD-L1 dependent manner. On the other hand, TREM-1^+^ TAMs recruited abundant CCR6^+^Foxp3^+^ Tregs into TME via CCL20/ERK/NF-κB pathway. Application of TREM-1 signaling inhibitor GF9 statistically decreased CCR6^+^Foxp3^+^ Tregs infiltration, increasing the therapeutic efficacy of ICBs, as a result postponed tumor progression (Wu et al., 2019). Aside from negatively regulating CD8^+^ T cells, TAMs also interfere the function of CD4^+^ T cells. It was reported that the mechanisms of glioma cells resistance to anti-PD-1 and anti-CTLA-4 were mediated by TAMs through PD-1/PD-L1/CD80 axis, accompanied by Treg accumulation. Co-culture of naïve CD4^+^ T cells with PD-L1-positive TAMs induced the expression of CD80 on CD4^+^T cells. PD-L1/CD80 interaction blockades antitumor immune responses to anti-PD-1 and anti-CTLA-4 therapies (Aslan et al., 2020).

Evidence from prostate cancer treated by ADT demonstrates that the interconnection between TAMs and CSCs can also lead to drug resistance. CSCs are capable of remodeling macrophages toward TAMs. Reciprocally, TAMs enhanced the stem-like characteristics of CSCs and drug resistance via IL-6/STAT3 signaling pathway (Huang et al., 2018). Consistent with this finding, in C57BL/6 mice model inoculated with MC38-CSCs (colon tumor) and 3LL-CSCs (lung cancer), TAMs generated milk-fat globule epidermal growth factor-VIII (MFG-E8) to regulate CSCs. MFG-E8 cooperated with IL-6 to stimulate STAT3 and hedgehog signaling pathways in CSCs, driving anticancer drug resistance (Jinushi et al., 2011). Moreover, the EMT induced by TAMs could be a potential mechanism amplifying peritoneal dissemination and causing chemoresistance in pancreatic cancer (Kuwada et al., 2018).

In conclusion, the mechanisms involved in antitumor drug resistance mediated by TAMs include release of multiple cytokines, transformation toward suppressive-M2 phenotype, suppressive effects on immune cells, modulation of CSC properties and promotion of EMT, which have been summarized in Table 2.

**TABLE 2:** Mechanisms of antitumor drug resistance mediated by TAMs.

Tumor type	Drug resistance	Mechanisms	References
Breast cancer	Paclitaxel	TAMs secreting cathepsins B and S.	Shree et al., 2011
	Cyclopamine	TAMs increasing the production of IL-6.	Xu X. et al., 2019
	Paclitaxel	TAMs secreting IL-10, activating STAT3 and elevating the expression of Bcl-2.	Yang et al., 2015
CRC	5-FU	TAMs secreting putrescine that inhibits the activation of JNK/caspase-3 pathway.	Zhang et al., 2016
	Oxaliplatin	TAMs secreting IL-6, activating IL-6R/STAT3/miR-204-5p pathway of tumor cells.	Yin et al., 2017
	Cisplatin	TAMs secreting MFG-E8 to regulate CSCs via activating STAT3 and hedgehog signal.	Jinushi et al., 2011
Gastric cancer	Cisplatin	TAMs producing exosomes containing miR-21, downregulating PTEN and enhancing PI3K/AKT.	Zheng et al., 2017
Glioma	Cisplatin	TAMs producing NO, inhibiting acid sphingomyelinase via NO/cGMp/PKA pathway.	Perrotta et al., 2018
	Anti-PD-L1/CTLA-4	PD-L1 of TAMs binding with CD80 of CD4^+^ T cells, inhibiting antitumor T cell responses.	Aslan et al., 2020
HCC	Anti-PD-L1	TREM-1^+^ TAMs damaging the cytotoxic functions of CD8^+^ T cells and causing CD8^+^ T cell apoptosis.	Wu et al., 2019
HNSCC	Gefitinib	TAMs secreting CCL15, activating CCL15/CCR1/NF-κB pathway.	Yin et al., 2019
Ovarian cancer	Cisplatin	TAMs secreting exosomes delivering miR-223, inactivating PI3K/AKT signal by targeting PTEN.	Zhu et al., 2019
Pancreatic cancer	Gemcitabine	TAM secreting pyrimidines competitively inhibits gemcitabine uptake and metabolism.	Halbrook et al., 2019
	Gemcitabine	TAMs upregulating cytidine deaminase, promoting drug degradation.	Weizman et al., 2014
	Gemcitabine	TAMs derived-exosomes transfer miR-365, upregulating cytidine deaminase.	Binenbaum et al., 2018
	Camptothecin	TAMs secreting IL-1β, promoting the production of COX-2 and PGE2 via ERK1/2.	Angst et al., 2008
	5-FU	TAMs inducing EMT.	Kuwada et al., 2018
Prostate cancer	ADT	TAMs enhancing the stem-like properties of CSCs via IL-6/STAT3 pathway.	Huang et al., 2018

*CRC, colorectal cancer; HCC, hepatocellular carcinoma; HNSCC, head and neck squamous cell carcinoma; 5-FU, 5-fluorouracil; ADT, androgen deprivation therapy; EMT, epithelial-mesenchymal transition.*

Furthermore, tumor-associated neutrophils (TANs) high infiltration within TME was related to tumor progression and drug resistance (Incio et al., 2016). Neutrophil polarization may affect their role in the tumor microenvironment. Similar to the classification of M1/M2 macrophages, TANs are sorted into antitumorigenic N1 phenotype and pro-tumorigenic N2 phenotype on the basis of their functional differences (Zhang Y. et al., 2020). TGF-β and type I IFN-γ were suggested to play essential roles in the polarization of TANs switching from N1 to N2 (Fridlender et al., 2009; Pylaeva et al., 2016). CXCL5 serves as a key factor in recruiting TANs (Nywening et al., 2018). Sorafenib administration led to increased secretion of CXCL5 derived from HCC cells, inhibiting TANs apoptosis by inducing HIF1 α/NF-κB/CXCL5 pathways, enhancing TAN infiltration in both animal models and HCC patients. Besides, HCC cells were able to polarize TANs and promote secretion of CCL2 and CCL17 by TANs through activating PI3K/Akt and p38/MAPK signals. As a response to HCC cells, TANs facilitate intra-tumoral infiltration of CCR2^+^macrophages and CCR4^+^Treg cells via CCL2-CCR2 and CCL17-CCR4 connections (Zhou S.L. et al., 2016). Therefore, combined blockade of TANs and TAMs present advantages in improving therapeutic outcomes (Nywening et al., 2018).

Generally, there are three approaches to target TAMs: reduced recruitment, direct depletion, and repolarization. CCL2 is a potent chemokine for monocytes recruitment and CCL2-CCR2 axis is the primary mediator for the expansion of macrophages in TME. In a PDAC mouse model, tumor cells-derived CCL2 inhibited the efficacy of ablative radiotherapy by recruiting inflammatory monocytes/macrophages into the TME to accelerate tumor proliferation and angiogenesis. While selective blockade of CCL2 using neutralizing antibodies interrupted the recruitment of monocytes/macrophages and produced synergistic antitumor effects with radiotherapy and prolonged survival time (Kalbasi et al., 2017).Next, colony-stimulating factor 1 receptor (CSF1R), receptor of CSF-1 and IL-34, is necessary for the proliferation, differentiation and survival of macrophages (Muñoz-Garcia et al., 2021). Therefore, avenues of targeting CSF-1R alone or combined with standard therapies seem to be feasible. Enhanced infiltration of TAMs was observed after radiotherapy in gliomas mouse model, which was responsible for tumor recurrence. While combined therapies of CSF-1R inhibitor, BLZ945, with radiotherapy significantly delayed glioma relapse. Particularly, CSF-1R could inhibit both resident-microglia and recruited monocyte-derived macrophage. Neutralizing antibody of CD49d could only inhibit the recruitment of monocyte-derived macrophage without effect on resident microglia. Compared to the CD49d neutralizing antibody, CSF-1R displays stronger efficacy in delaying relapse (Akkari et al., 2020). Nevertheless, existence of antigen presenting cells (APCs) including macrophages and dendritic cells (DCs) are essential for T cells activation. Elimination of macrophages could reduce the efficacy of immunotherapy (Diggs et al., 2020). Repolarization of TAMs by phenotype switch from M2 to M1 provides an opportunity to balance immune microenvironment, and compensates for the disadvantages of depletion of all macrophages. In 4T1 breast cancer mouse model, selectively targeting M2 TAMs, by simultaneously delivery of CSF-1R and CD47/SIRPα inhibitors via dual-inhibitor-loaded nanotherapeutic technique, results in increased M2 repolarizations into M1, enhanced phagocytosis activity, and satisfactory anticancer outcome (Ramesh et al., 2019). This preclinical trial suggests a promising and preferable strategy of targeting TAM.

### Myeloid-Derived Suppressor Cells (MDSCs)

Myeloid-derived suppressor cells are an immature heterogeneous population of the myeloid family, which contain two subsets of cells. One group is defined as granulocytic-MDSCs (G-MDSCs), with the CD11b^+^Ly-6G^+^Ly-6C^low^ phenotype, expressing high levels of arginase-1 (Arg-1). This population are also termed as PMN-MDSC due to their polymorphonuclear (PMN) morphology. The other group is identified as monocytic-MDSCs (Mo-MDSCs) because of their monocytic-like morphology, with the CD11b^+^Ly-6G^low^Ly-6C^hi^ marker (Youn et al., 2008; Talmadge and Gabrilovich, 2013).

**FIGURE 3:**
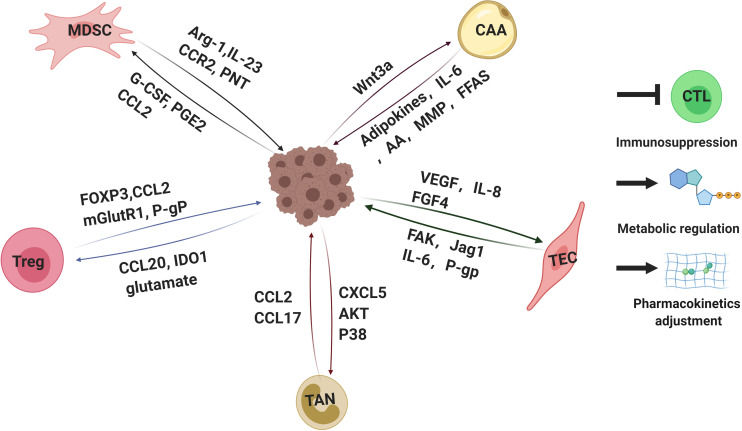
Interactions of tumor cells with parts of non-malignant cells within tumor microenvironments mediating drug resistance. Tumor microenvironment contains quantities of non-malignant stromal cells which are essential for tumor progression and drug resistance. Interactions between tumor cells and stromal cells polarize stromal cells. In turn, the stromal cells educated by tumor cells secret a variety of molecules, leading to immunosuppression, metabolic regulation and pharmacokinetics adjustment, which eventually weakens therapeutic efficacy. Arg-1, arginase-1; IL-23,interlukin-23; PNT, peroxynitrite; G-CSF, granulocyte-colony stimulating factor; PGE2, prostaglandin E2; FOXP3, forkhead box protein P3; mGlutR1, glutamate receptor 1; P-gP: P-glycoprotein, IDO1, indoleamine 2,3-dioxygenase; CAA: cancer associated adipocyte; FFAs: free fatty acids; AA: arachidonic acid; VEGF: vascular endothelial growth factor; FGF4: fibroblast growth factor 4; FAK: focal adhesion kinase. CTL, cytotoxic T lymphocytes. The figure was created with BioRender.com.

One of the main features of MDSCs is immunosuppression (Figure 3). MDSCs lead to the inhibition of different types of immune cells, but the primary targets of MDSCs are T cells. The factors participated in MDSCs-mediated immunosuppression including Arg-1, IL-23, IL-10, TGF-β, PGE2, and many others (Gabrilovich, 2017; Xu et al., 2017). Recent studies elucidated that the reciprocal action between malignant cells and MDSCs plays a critical role in immunosuppressive chemoresistance. The level of PMN-MDSC and Arg-1 in serum of multiple melanoma (MM) patients was higher than that of healthy individuals. Furthermore, PMN-MDSC extracted from MM patients could suppress the activation of T cells effectively and protect MM cells from bortezomib treatment. However, this protection was offset by Arg-1 inhibitor (Romano et al., 2018). Mechanically, MM cells survival induced by MDSCs is dependent on AMPK activation, MCL-1 and BCL-2 expression in myeloma cells (De Veirman et al., 2019). In addition, it has been elucidated that the acquired ADT resistance of prostate cancer could be blamed on IL-23 secreted by MDSCs. IL-23-IL-23R-RORγ axis activated the androgen receptor pathway in prostate cancer cells, regulating resistance to ADT (Calcinotto et al., 2018).

Besides the mechanisms mentioned above, tumor cells are found to recruit and activate MDSCs in TME under therapeutic pressure. *In vitro* and *in vivo* results as well as clinical samples demonstrated that the expression of granulocyte-colony stimulating factor (G-CSF) in cervical cancer cells was significantly linked with MDSC infiltration and chemoresistance (Kawano et al., 2015). Blockade of colony stimulating factor-1 receptor (CSF-1R) can prevent immune resistance to immunotherapy (Holmgaard et al., 2016). Doxorubicin resistant breast cancer cells escape immune surveillance by secreting PGE2. PGE2 then bind with its receptor on MDSCs, EP2/EP4, leading to accumulation of MDSCs. PGE2/miR-10/AMPK signaling pathway of MDSCs was activated, with inhibition of CD4^+^CD25^–^ T cells and decreased production of IFN-γ (Rong et al., 2016). BRAF inhibitor-resistant melanomas induced expansion of CCR2-expressing monocytic-MDSCs in the TME by producing CCL2, activating MDSCs through MAPK signaling. Combination of checkpoint blockade with MDSC depletion significantly reversed BRAF inhibitor resistance (Steinberg et al., 2017). Of note, MDSCs not only play a part in functionally suppression of T cells, but also participate in (cytotoxic T lymphocytes) CTL-mediated cytolysis. Lu et al. (2011) demonstrated that MDSCs are a predominant origin of the free radical peroxynitrite (PNT) in lung, pancreatic, and breast cancer samples. PNT induces post-translational modifications of cell surface molecules while not affecting cell viability, inhibiting processed peptides presentation to tumor-associated MHC molecules. Thus, tumor cells escape from antigen-specific CTLs and acquire immunological resistance (Lu et al., 2011).

These findings suggest the important role of MDSCs (Table 3), which motivate us to consider them as a target to overcome tumor resistance. MDSCs can be diminished by using chemotherapeutic drugs. CD33 is identified as a pathological marker for recognizing MDSCs across cancer subtypes and is responsible for immunosuppressive microenvironment. Conjugating anti-CD33 antibody with toxin calicheamicin, so called as gemtuzumab ozogamicin, can deplete CD33^+^ MDSCs thus to restore T cell activity and reactive CAR-T cell response (Fultang et al., 2019). Meanwhile, this strategy shows good results in clinical trials (Lancet et al., 2020; Schlenk et al., 2020). Another approach to inhibit MDSCs is to block their signaling cascades. IL-1β is an upstream mediator of myeloid resistance. Anti-IL-1β significantly decrease the infiltration of PMN-MDSCs and TAMs in TME. The combined use of anti-IL-1β and anti-PD-1 or cabozantinib increased anti-tumor activity (Aggen et al., 2021). One of the most promising techniques for targeting MDSCs is to promote their reprogramming. Increased activation of PKR-like endoplasmic reticulum kinase (PERK) in MDSC contribute to MDSC-mediated T cell dysfunction by stimulating transcriptional factor NRF2. Inhibition of PERK interrupts cytosolic mitochondrial DNA-STING-type I IFN axis and converts MDSCs into normal myeloid cells with activated CTL immunity. PERK inhibitor combined with anti-PD-L1 therapy shows synergetic effects in B16 bearing mice (Mohamed et al., 2020).

**TABLE 3:** Mechanisms of antitumor drug resistance mediated by MDSCs.

Tumor type	Drug resistance	Mechanisms	References
Breast cancer	Doxorubicin	Cancer cells secreting PGE2 and activating PGE2/miR-10/AMPK signals within MDSCs.	Rong et al., 2016
Cervical cancer	Platinum-based chemotherapy	Cancer cells expressing G-CSF, causing MDSCs accumulation and T cell suppression.	Kawano et al., 2015
HCC	5-FU, ADM	Cancer cells-derived IL-6 enhancing the activity and expansion of MDSCs.	Xu et al., 2017
Melanoma	PLX4720	Melanomas inducing expansion of CCR2-expressing monocytic-MDSCs in the TME by producing CCL2, activating MDSCs through MAPK signaling.	Steinberg et al., 2017
MM	5-FU, bortezomib	MDSCs inducing AMPK activation and MCL-1 and BCL-2 expression in myeloma cells.	De Veirman et al., 2019
Prostate cancer	Castration-resistance	IL-23 secreted by MDSCs activating IL-23-IL-23R-RORγ axis of androgen receptor pathway in prostate cancer cells.	Calcinotto et al., 2018

*PGE2, prostaglandin E2; G-CSF, granulocyte-colony stimulating factor; HCC, hepatocellular cancer; 5-FU, 5-fluorouracil; ADM, adriamycin; MM, multiple myeloma.*

### T-Regulatory Lymphocytes (T-Reg)

Tregs are a subpopulation of immunosuppressive T cells identified as CD4^+^CD25^+^, and are characterized by expression of the Foxp3, which is essential for Tregs development and differentiation. In the TME, increased infiltration of Tregs has been defined to positively associate with poor prognosis and chemoresistance in melanoma, glioblastoma, HNSCC, ovarian, colorectal, renal and lung cancer (Bamias et al., 2008; Li et al., 2012; Schuler et al., 2013; Liu X.D. et al., 2015; Wang D. et al., 2019; Imbert et al., 2020; Long et al., 2020). Acquired resistance to ICBs remains a challenge in cancer therapy as a compensatory pathway emerges in TME to evade the antitumor effects induced by ICBs. Apart from the induction of MDSCs we summarized above, increased ratio of Tregs within TME suggests another cellular mechanism (Figure 3).

*In vivo*, 5-FU treatment elevated the expression of CCL20 in colorectal cancer cells (CRC) by activating FOXO1/CEBPB/NF-κB signaling, which promoted the recruitment of Tregs within TME. The proportion of CD4^+^Foxp3^+^ Tregs in tumor infiltrating lymphocytes (TIL) of CRC was dramatically higher than that of paired controls. Expression of Foxp3^+^ was obviously associated with resistance-related genes, including BCL2, WNT1, ATP8A2 and VIM. CCL20 blockers inhibit tumor progression and restore sensitivity to 5-FU of CRC (Wang D. et al., 2019). *In vitro*, mouse Lewis lung cancer (LLC) cells, overexpression of Foxp3^+^ was also identified to increase the IC50 values of Adriamycin (ADM) and mitomycin C (MMC) (Li et al., 2012). Moreover, recent evidences have proven that amino acid metabolism could affect immune cell response to tumor cells by regulating Tregs. Folgiero et al. (2016) disclosed that mTOR inhibitor, rapamycin, induces indoleamine 2,3-dioxygenase (IDO1) expression in medulloblastoma cells. IDO1, one of three enzymes catalyzing the degradation of TRP along the kynurenine pathway, led to Treg expansion by inducing CCL2 expression, creating an immune-tolerance microenvironment. VEGF inhibition is a critical method for inhibiting angiogenesis. While results showed that resistance to VEGF inhibitors is elicited by upregulation of Tregs. Specifically, the increased expression of glutamate/cystine antiporter SLC7A11/xCT causes elevated extracellular glutamate in the VEGF inhibitors treated glioblastoma cells, promoting Tregs proliferation, activation and suppressive function (Long et al., 2020). Indeed, high levels of ATP metabolism within TME have been determined to be linked with Tregs as well as immunosuppression (Deaglio et al., 2007). Experiments conducted by Gourdin et al. (2018) showed that CD73^+^CD4^+^ effector T cells (Teffs) act synergistically with CD39^+^Tregs to metabolize ATP to immunosuppressive adenosine, restraining the function of Teffs on IL-17A production. What is more, the compensatory mechanisms between CSF1R^+^ TAMs and Foxp3^+^ Treg cells promote resistance to tumor immunotherapeutic agents. Either reduction of CSF1R^+^ TAMs or Foxp3^+^ Tregs will limit the function of CD8^+^ T cells on tumor growth. Only co-blockade of CSF1R^+^ TAMs and Foxp3^+^ Tregs can obtain a synergistic effect on augmentation of CD8^+^ T cells and tumor suppression (Gyori et al., 2018).

Collectively, over time and as a result of stress selection, some tumors augment Tregs function by producing more self-antigens and acquire compensatory pathways to evade the antitumor therapy, as well as resistance to ICBs (Table 4). Therefore, a deeper comprehension of the mechanisms and molecular pathways by which ICBs regulate T cells and Tregs, is necessary to design better immunotherapeutic strategies. Therapeutic approaches depleting Tregs mainly focused on their molecular markers, such as CD25, CCR8. Treg depletion using anti-25 antibody combined with dual immunotherapy and radiotherapy restored antitumor immunity durably and induced immunologic memory (Oweida et al., 2018). Fc-optimized anti-human CCR8 antibody results in restrict Treg exhaustion without influence on Teffs (Campbell et al., 2021). CCR8-directed Treg elimination shows synergistic effect with PD-1 blockade in mouse model (Van Damme et al., 2021). In addition, CCR4 is a robust chemokine receptor for Treg recruitment in TME. Anti-CCR4 mAb (mogamulizmab) is under clinical investigation either as a monotherapy or combinational choice of anti-PD-1 mAb (Kurose et al., 2015; Doi et al., 2019).

**TABLE 4:** Mechanisms of antitumor drug resistance mediated by Tregs.

Tumor type	Drug resistance	Mechanisms	References
CRC	Immunotherapy resistance	PI3Kδ-driven Foxp3^+^ Treg cells compensating CSF1R^+^ TAMs to limit the function of CD8^+^ T cells.	Gyori et al., 2018
	5-FU	CCL20 secreted by colorectal cancer cells activating FOXO1/CEBPB/NF-κB signaling, promoting the recruitment of Tregs.	Wang D. et al., 2019
Glioblastoma	VEGF mAb	Expression of glutamate/cystine antiporter SLC7A11/xCT causing elevated extracellular glutamate in glioblastoma cells, promoting Tregs proliferation.	Long et al., 2020
LLC cells	ADM, MMC	Foxp3 upregulating the expression of mdr1 mRNA and P-gp, reducing the sensitivity of LLC cells to ADM and MMC.	Li et al., 2012
Medulloblastoma	Rapamycin	IDO1 expression in medulloblastoma cells causing Treg expansion by CCL2 mediator.	Folgiero et al., 2016

*CRC, colorectal cancer; TAM, tumor associated macrophage; 5-FU, 5-fluorouracil; VEGF, vascular endothelial growth factors; mAb, monoclonal antibody; LLC, Lewis lung cancer; ADM, adriamycin; MMC, mitomycin C; mrd1, multidrug resistance protein 1; P-gp, P-glycoprotein; IDO1, indoleamine 2,3-dioxygenase 1.*

In summary, due to the presence of TAMs, MDSCs, Tregs and their related factors, immunosuppressive microenvironments are now considered a major obstacle to ICBs. A range of investigations on turning non-immunogenic tumors into immunogenic tumors have been developed, for example, combinations of ICBs with anti-angiogenic agents (Makker et al., 2019), targeted therapies (De Henau et al., 2016), and other checkpoint inhibitors (Bedke et al., 2021). These therapeutic strategies should maximize the effectiveness of immunotherapy and induce sustained anti-tumor immune responses, to reach better clinical outcomes and response rates in cancer patients.

## Mesenchymal Stem Cells (MSCs)

Mesenchymal stem cells (MSCs), commonly called mesenchymal stromal cells, are one of another pivotal components of the TME (Shi et al., 2017). MSCs have been reported to be involved in many distinct steps of tumorigenesis, such as angiogenesis, metastasis, epithelial-mesenchymal transition, anti-apoptosis, pro-survival, immunosuppression and therapy resistance. A growing body of researches suggest the important role of MSCs in drug resistance. MSCs inherently possess the ability to be chemoresistance and can confer this resistance to many types of cancer cells, including osteosarcoma, acute lymphoblastic leukemia, breast cancer, ovarian cancer, oral squamous cell carcinoma, hepatocellular carcinoma and so on (Roodhart et al., 2011; Han et al., 2014; Ji et al., 2015; Xu et al., 2018; Lu et al., 2020; Raghavan et al., 2020; Wang S. et al., 2020). Apart from bone marrow, where MSCs were originally discovered, adipose tissue, peripheral blood, umbilical cord are also important sources of MSCs. As a heterogeneous group of progenitor cells, MSCs are known for the capacity to transdifferentiate into multicellular lineages including chondrocytes, osteocytes, adipocytes, myocytes, astrocytes, fibroblasts, and pericytes (Shan, 2004). Featured for their ability to adhere to the plastic, MSCs are also typically positive for certain patterns of surface markers (CD73, CD105, CD44, CD29, and CD90), concomitantly they lack expression of endothelial markers (CD34, CD31, and vWF) and hematopoietic markers (CD34, CD45, and CD14) (Reagan and Kaplan, 2011).

**FIGURE 4:**
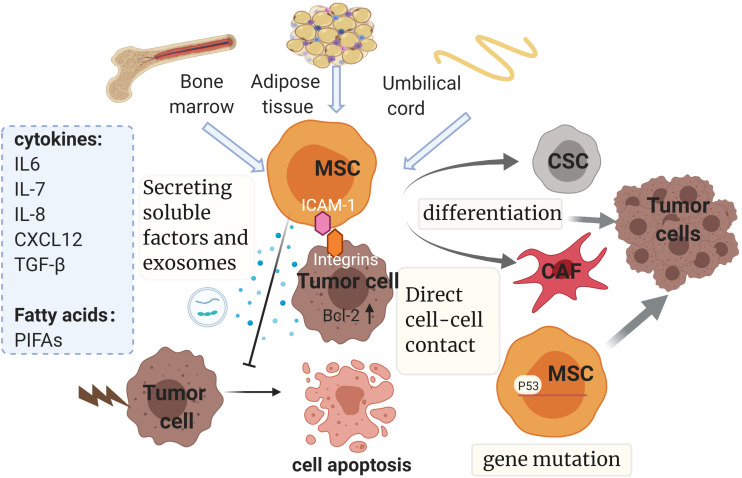
Mesenchymal stem cells and drug resistance. Mainly isolated from bone marrow, adipose tissue and umbilical cord, MSCs promote drug resistance in multiple ways, such as secreting soluble factors and exosomes, genetic mutations, direct cell-cell contact and differentiating into CAF or CSC. The diverse soluble factors and exosomes derived from MSCs could induce chemoresistance dependent on multiple pathways and downstream mechanisms, some of which relate to the inhibition of tumor cell apoptosis by regulating the expression of apoptosis-related proteins. It is noteworthy that chemoresistance properties of MSCs can be conferred via direct cell–cell contact with tumor cells as well. This interaction can enhance the expression of Bcl-2. Also, gene mutations such as p53 deficiency in MSCs could contribute to drug resistance by mediating antitumor immunity. Finally, MSCs have the potential to evolve into CAFs and CSCs, which have been reported to be involved in the development of resistance. MSC, mesenchymal stem cell; CSC, cancer stem cell; CAF, cancer-associated fibroblast; SDF-1: stromal cell-derived factor-1; TGF-β, transforming growth factor-β; PIFAs, platinum-induced fatty acids. ICAM-1, intercellular adhesion molecule-1. The figure was created with BioRender.com.

According to recent studies, MSCs can promote drug resistance in different ways as follows (Figure 4): (1) secreting soluble factors; (2) delivering MSC-exosomes; (3) genetic mutations in MSCs; (4) direct cell-cell contact; (5) differentiating into CAF or CSC.

### Secreting Soluble Factors

Mesenchymal stem cells can release diverse growth factors, cytokines and fatty acids which are involved in multiple pathways and downstream mechanisms, leading to the acquisition of drug resistance (Scherzed et al., 2011; Luo et al., 2014; Senthebane et al., 2017). As discussed earlier, increased IL-6 secretion by CAFs was described to induce chemoresistance. Similarly, MSCs derived IL-6 plays an important role in the resistance via the activation of downstream STAT3 cascades in different tumors. For example, in breast cancer, MSCs secreting IL-6 causes protection of MCF-7 cells from cisplatin-induced apoptosis, which is mediated by activating STAT3 signaling pathway with the markedly decreasing expression of Bax (Xu et al., 2018). In osteosarcoma cells, IL-6 produced by MSCs activates the JAK2/STAT3 signaling, with the subsequent up-regulation of multidrug resistance protein (MRP) and P-gP expression, which is relevant to poor response rates of clinical osteosarcoma chemotherapy (Tu et al., 2016). And in NPC, MSC-derived IL-6 was found to significantly upregulate STAT3 signal pathway, which could bind to the promoter region of NET5 gene, thus contributing to the increased expression of CD73. Subsequently, the elevated expression of CD73 promotes NPC tumor growth and inhibition of cisplatin-induced apoptosis, mediating resistance of NPC cells to cisplatin (Zeng et al., 2020). In head and neck carcinoma, other paracrine cytokines secreted by MSCs, including IL-6, IL-7, IL-8, and growth factors such as VEGF, have also been reported to be involved in the development of tumor drug resistance (Scherzed et al., 2011). IL-7 was demonstrated to be highly expressed by MSCs in chronic myeloid leukemia (CML). It is known that imatinib (IM) and nilotinib (NI) are remarkably effective therapeutic agent of BCR/ABL-positive CML and have improved the clinical outcomes. The IL-7-mediated JAK1/STAT5 pathway bypasses BCR/ABL signals, protecting tumor cells from imatinib (IM) and nilotinib (NI) in a BCR/ABL-independent way. Interestingly this could explain the early resistance before the BCR/ABL genetic mutation (Zhang et al., 2019). IL-8, a neutrophil chemoattractant, is one of the CXC chemokine receptors 1 and 2 (CXCR1/2) ligands. It has been reported that through highly releasing chemokine (C-X-C motif) ligand 1 (CXCL1), CXCL2, and IL-8, MSCs manipulate metabolic programs to promote M2 macrophages differentiation, leading to abolition of their tumoricidal functions, thus facilitating the acquisition of chemoresistance in ovarian tumor cells. As expected, CXCR1/2 inhibitor reparixin could revert MSC-mediated drug resistance in ovarian cancer by restoring the anti-tumoral function of macrophages both *in vitro* and *in vivo*. Hence, reparixin has the potential to improve the efficacy of carboplatin in cancer cells (Le Naour et al., 2020).

Another chemokine CXCL12, also named SDF-1, is constitutively secreted by MSCs. SDF-1a/CXCR4 interactions between CML cells and MSCs protect CML cells from imatinib-induced cell death by reducing activation of caspase-3 and promoting the expression of Bcl-XL, an important anti-apoptotic protein inhibited by imatinib (Vianello et al., 2010). SDF-1a/CXCR4 axis was also reported to mediated drug resistance in acute lymphoblastic leukemia (ALL). MSCs increase the secretion of CXCL12, accompanied with the elevated expression of surface CXCR4 on chemo-resistant leukemic cells in the presence of vincristine. Furthermore, vincristine induced ALL apoptosis can be attenuated by upregulating Bcl-2 and downregulating Bax. Interestingly, all of these could be effectively reversed by the CXCR4 antagonist, AMD3100, which blocks the SDF-1a/CXCR4 axis and then restores sensitivity of ALL to chemotherapy (Wang S. et al., 2020). In addition, MSCs derived SDF-1a was also noted to mediate the cross-talk with the ovarian cancer cells, thus inducing their thermotolerance from hyperthermia intraperitoneal chemotherapy, a selective therapeutic approach for ovarian carcinoma (Lis et al., 2011).

Fatty acids are another important factor released by MSCs. Researches have demonstrated that the MSCs induced chemoresistance could be mediated by secreting two unique platinum-induced fatty acids (PIFAs). These two distinct PIFAs, namely 12-oxo-5,8,10-heptadecatrienoic acid (KHT) and hexadeca-4,7,10,13-tetraenoic acid [16:4(n-3)] are reported to confer resistance to a broad spectrum of anticancer drugs in minor quantities. Interestingly, inhibition of the central enzymes (cyclooxygenase-1 and thromboxane synthase), associated with the production of PIFAs, significantly enhances the chemotherapy efficacy (Roodhart et al., 2011). While the exact mechanism by which MSCs derived PIFAs induce chemoresistance remains to be elucidated.

Apart from cytokines, chemokines and fatty acids, the expression of growth factors like TGF-β was also reported to be upregulated in MSCs. In hepatocellular carcinoma (HCC), the tumor inflammatory microenvironment stimulates the overexpression of TGF-β in MSCs, inducing chemoresistance of HCC cells by promoting autophagy. Knockdown the expression of TGF-β by transfecting siRNA attenuates the ability of MSCs to induced autophagy and restore the sensitivity to chemotherapy in HCC cell (Han et al., 2014). In gastric cancer (GC), evidence presents that MSCs secreted TGF-β1 promotes fatty acid oxidation (FAO) to support stemness features and drug resistance, which is mediated by activating SMAD2 and SMAD3 and promoting lncRNA MACC1AS1 expression (He et al., 2019).

### Delivering MSC-Exosomes

Exosomes transferring a variety of bioactive molecules including mRNAs, miRNAs and proteins, play important roles in inter- and intra-cellular interplay through pathways that influence TME regulation and drug resistance (Zhang X. et al., 2017; Adamo et al., 2019). Accumulating evidence suggests that MSC-derived exosomes contribute to chemoresistance through different ways, including drug sequestration, delivering specific mRNA molecules and proteins, mediating communication between MSCs and cancer cells (Adamo et al., 2019).

Mesenchymal stem cell-derived exosomes are reported as important mediators of drug resistance in gastric cancer. MSC-exosomes incorporate into the gastric cancer cells, stimulating the activation of CaM-Ks (predominantly CaM-KII and CaM-KIV), triggering downstream Raf/MEK/ERK signaling cascade activation, which consequently upregulates the expression of multi-drug resistance associated proteins including MDR, MRP, and LRP, eventually conferring chemoresistance in gastric cancer (Ji et al., 2015). MSC-derived exosomes could deliver the contents to cancer cells, and this interaction is responsible for drug resistance. For instance, in breast cancer, MSCs released exosomes contain miR-222/223, entering breast cancer cells (BCCs) to promote the transition into cycling quiescence, which plays a key role in drug evasion. Further study observed that delivering anti-miR-222/223 by antagomiR-loaded MSCs to the dormant BCCs improves the efficiency of carboplatin and then increased host survival (Bliss et al., 2016). Moreover, MSC-exosomes mediated drug resistance has been documented in hematological tumors as well. For example, bone marrow stromal cells (BMSCs) derived exosomes function as a communicator mediating cell-to-cell communication through transference of their contents in multiple myeloma (MM) cells. BMSC-derived exosomes activate several pathways including p38, p53, c-Jun N-terminal kinase, and AKT pathways related to survival, promoting MM cell survival and chemoresistance *in vivo* by increasing antiapoptotic protein Bcl-2 and reducing the cleavage of caspase-9, caspase-3 (Wang et al., 2014).

### Direct Cell-Cell Contact

In addition to releasing paracrine factors and exosomes, MSCs could confer resistance through direct cellular interactions. This cross-talk between cancer cells and MSCs enables to promote the activation of a series of signaling cascades in different tumors (Wang J. et al., 2018). Mudry et al. (2000) has demonstrated that MSCs protect leukemia cells from cytarabine and etoposide cytotoxicity by directly contacting with leukemic cells, thus promoting the expression of vascular cell adhesion molecule-1 (VCAM-1). Further investigations have been done to figure out the detailed protective mechanisms of MSCs adhesion. In T cell acute lymphoblastic leukemia (T-ALL), MSCs adherence to cancer cells, mediated by intercellular adhesion molecule-1 (ICAM-1), is proved to induce mitochondria transfer between MSCs and T-ALL cells, leading to the decrease of mitochondrial intracellular ROS levels in T-ALL cells. It is well accepted that excess ROS is responsible for cell apoptosis, which is one of major mechanisms for chemotherapy (Wang J. et al., 2018). Noteworthily, treating with blocking antibody against ICAM-1 could effectively reduce adhesion between T-ALL cells and MSCs, reducing mitochondrial transfer, thereby restoring chemotherapy sensitivity. Additionally, MSCs protect CML stem/progenitor cells from TKI induced apoptosis by increasing N-Cadherin-mediated adhesion and enhancing transcription of β-catenin target genes in primary CML CD34^+^ cells. Not surprisingly, the β-catenin inhibitor, ICG001, targeting Wnt signaling enhanced the sensitivity of CML to TKI cocultured with MSC (Zhang et al., 2013).

Aside from hematological malignancies, MSCs are also elucidated to be involved in the drug resistance in solid tumors by direct contact. In oral squamous cell carcinomas (OSCCs), co-cultured with MSCs, the human derived OSCC cell lines are shown activation of PDGF-AA/PDGFR-α autocrine loop, accompanied with the upregulation of AKT, which reduced apoptotic response by increasing expression of Bcl-2 and eventually contributed to cisplatin resistance in OSCC (Wang J. et al., 2020). MSCs co-culture also improves stemness and confers chemo-resistance in gastric cancer (GC) cells by inducing the expression of metabolic pathways related LncRNA histocompatibility leukocyte antigen complex P5 (HCP5) in GC cells. Mechanistically, HCP5 is confirmed to target miR-3619-5p, facilitating FAO by activating AMPK/PGC1α/CEBPB axis to promote stemness and concomitantly mediate chemoresistance in GC cells (Wu et al., 2020).

### Genetic Mutations in MSCs

It has long been acknowledged that somatic mutations in tumor cells is a major cause of drug resistance. Some recent studies found genomic alterations also occur in non-malignant cells, which contribute to latent tumor recurrence in patients receiving chemotherapy and radiotherapy (Shi et al., 2017). Patocs et al. (2007) observed genetic mutations in breast cancer stromal cells partly contribute to the poor clinical outcome. In B16F0 melanoma cells, p53-deficient MSCs increase the expression of inducible nitric oxide synthase (iNOS), which is known to be responsible for regulating antitumor immunity and cell survival, leading to vigorous immunosuppression, promoting the facilitation of tumor growth, thus conferring therapy resistance. The inhibition of iNOS could reverse the enhanced tumor-promoting effects of p53-deficient MSCs, suggesting a window of opportunity to target iNOS to restore the sensitivity of MSCs (Huang et al., 2014).

### Differentiating Into CAF or CSC

As mentioned before MSCs have the potential to differentiate into multicellular lineages and their conversion to CSCs or CAFs could be another mechanism to confer therapeutic resistance. CSCs have the ability to induce tumor progression and metastasis, and are considered to be inherently resistant to chemotherapy. It has been shown that targeting the methylation of two tumor suppressor gene HIC1 and RassF1A in MSCs induces their differentiating into CSCs and obtaining CSCs characteristics, including the acquisition of drug resistance along with loss of anchorage dependence, increasing colony-forming capacity and pluripotency (Teng et al., 2011). CAFs, as discussed above, play critical role in drug resistance. Some studies have suggested human BM-MSC from healthy donors can transform toward αSMA- and FSP-1-expressing CAFs when exposed to the conditioned medium of tumor cells (Mishra et al., 2008). Dzobo et al. (2016) observed that longer incubation of breast cancer MDAMB 231 cells with human MSCs promotes hMSCs differentiate into CAF, along with the increasing levels of α-SMA and type I collagen, which is mediated by TGF-β/Smad signaling pathway (Senthebane et al., 2017). Further investigation reveals the relation between CAF and MSC in human cancer and their common contribution to drug resistance. Through analyzing human neuroblastoma (NB) tumor, Borriello et al. demonstrated a group of αFAP- and FSP-1-positive CAF cells that harbor similar phenotype and functionality to BM-MSC in the tumor stroma. These cells (designated CAF-MSC) contribute to various steps of tumor development, ranging from enhancing cell proliferation, decreasing cell apoptosis to endowing resistance to chemotherapy. Further study demonstrated that pro-tumorigenic effects mediated by CAF-MSC are associated with the activation of STAT3 and ERK1/2 in NB cells, consistent with IL-6 induced drug resistance mentioned above (Borriello et al., 2017). Hence the inhibition of STAT3 and ERK1/2 may open a new window to enhance the chemotherapy sensitivity.

Based on these multiple mechanisms of MSCs mediated drug resistance (Table 5), targeting the secreting of paracrine factors such as CXCR1/2, CXCL12 and PIFAs is proved to be a novel therapeutic approach (Le Naour et al., 2020; Wang S. et al., 2020). Moreover, due to the multifaceted role of MSCs in tumor progression, MSC-based anti-cancer therapies guide new strategies to overcome therapy resistance. For example, MSCs are being exploited as selective carriers for drug delivery in cancers because of their strong tropism and recruitment to tumors (Li et al., 2021). Several preclinical studies have been conducted to exploit the tumor-targeting ability of MSCs, using MSCs as an excellent *in vivo* vehicle to deliver tumor-killing drugs (Kim et al., 2015; Yin et al., 2018). Further therapeutic strategy is genetic modification of MSC to overexpress specific immunomodulatory cytokines, suicide genes and other molecules that can effectively inhibit tumor progression, including IFNα, IFNβ and (TNF)-related apoptosis-inducing ligand (TRAIL) (Kidd et al., 2010; Kim et al., 2012). Phase I and Phase II clinical trials of MSC-based anti-cancer therapies are currently in progress (von Einem et al., 2019). In conclusion, an in-depth exploration of the cellular and molecular mechanisms of drug resistance in MSCs is essential to improve existing anticancer therapies.

**TABLE 5:** Mechanisms of antitumor drug resistance mediated by MSCs.

Tumor type	Drug resistance	Mechanisms	References
ALL	Cytarabine, etoposide	T-ALL cell/MSC adhesion mediating mitochondria transferring.	Wang J. et al., 2018
	Vincristine	MSCs secreting CXCL12, binding to surface CXCR4 on ALL cells, mediating apoptosis rate, and apoptosis-related protein expression.	Wang S. et al., 2020
Breast cancer	Platinum	MSCs secreting two unique PIFAs.	Roodhart et al., 2011
	Carboplatin	MSCs releasing exosomes containing miR-222/223.	Bliss et al., 2016
	Cisplatin	MSCs-derived IL-6 activating STAT3 pathway with the markedly decreasing expression of Bax.	Xu et al., 2018
CML	Imatinib	MSCs secreting CXCL12, reducing activation of caspase-3 and promoting the expression of Bcl-XL.	Vianello et al., 2010
	TKI	MSCs increasing N-Cadherin-mediated adhesion and enhancing transcription of β-catenin target genes in primary CML CD34^+^ cells.	Zhang et al., 2013
	TKI	MSCs releasing IL-7, mediating JAK1/STAT5 pathway, activating bypassed BCR/ABL signals.	Zhang et al., 2019
Gastric cancer	5-FU	MSC-exosomes activating CaM-Ks, triggering downstream Raf/MEK/ERK signaling cascade activation.	Ji et al., 2015
	5-FU, oxaliplatin	MSCs secreting TGF-β1, promoting FAO to support stemness features and drug resistance.	He et al., 2019
	5-FU, oxaliplatin	MSC-induced lncRNA HCP5 driving FAO through miR-3619-5p/AMPK/PGC1α/CEBPB axis.	Wu et al., 2020
HCC	Cisplatin	The overexpression of TGF-β in MSCs promoting autophagy.	Han et al., 2014
Multiple myeloma	Bortezomib	MSC-exosomes activating several pathways including p38, p53, c-Jun N-terminal kinase, and AKT pathways related to survival.	Wang et al., 2014
NPC	Cisplatin	MSC-derived IL-6 upregulating STAT3 signal pathway, increasing expression of CD73.	Zeng et al., 2020
Osteosarcoma	Doxorubicin	MSC-derived IL-6 activating the JAK2/STAT3 signaling, with the subsequent up-regulation of MRP and P-gP expression.	Tu et al., 2016
Ovarian cancer	Carboplatin	MSCs highly releasing CXCL1/2 and IL-8, promoting M2 macrophages differentiation.	Le Naour et al., 2020
HNSCC	Paclitaxel	MSCs secreting various cytokines in a paracrine manner.	Scherzed et al., 2011

*MSCs, mesenchymal stem cells; ALL, acute lymphoblastic leukemia; PIFAs, platinum-induced fatty acids; CML, chronic myeloid leukemia; TKI, tyrosine kinase inhibitors; 5-FU, 5-fluorouracil; FAO, fatty acid oxidation; lncRNA, long non-coding RNA; HCC, hepatocellular carcinoma; NPC, nasopharyngeal carcinoma; MRP, multidrug resistance protein; P-gp, p-glycoprotein; HNSCC, head and neck squamous cell carcinoma.*

## Cancer Associated Adipocytes

Clinical data have shown epidemiological evidence linking obesity and poor prognosis in cancers at multiple specific sites (Calle et al., 2003). Growing researches highlight the independent effect of obesity to induce resistance to chemotherapies especially in breast cancer, ovarian cancer, prostate cancer, and leukemia (Ewertz et al., 2011), all of which implicate the underlying role of adipocytes in tumor drug resistance. Adipocytes are key components as one of stromal cells in TME (Nieman et al., 2013). It is noteworthy that the bidirectional cross-talk between adipocytes and cancer cells may partly explain cancer progression. Co-cultured with cancer cells, adipocytes are observed to exhibit remarkable phenotypic changes, designated as cancer associated adipocytes (CAAs) (Muller, 2013). And these phenotypic and functional alterations including the delipidation, reduced expression of adipose markers such as Ap2(FABP4), adiponectin and the overexpression of inflammatory cytokines are found to be related to drug resistance (Dirat et al., 2011). Bochet et al. (2013) demonstrated Wnt3a secreted by tumor cells could contribute to the alteration of adipocyte. Emerging evidence indicates that CAAs in TME play dynamic and sophisticated roles in determining resistance to treatments through distinct mechanisms involving regulating metabolism, secreting various factors, altering chemotherapy pharmacokinetics, and remodeling extracellular matrix (Muller, 2013; Choi et al., 2018; Zhang M.M. et al., 2018; Lehuédé et al., 2019).

### Regulating Metabolism

Adipocytes were initially identified as a tremendous energy storage providing high-energy metabolites. It is natural to expect a metabolic cross-talk between adipocytes and tumor cells contributing to the progression of tumor cells (Figure 3). The “Warburg effect” and “reverse Warburg effect” have been shown to be responsible for the drug resistance mediated by CAFs, and this concept could be applied to adipocytes as well (Duong et al., 2017). Under hypoxic conditions, lactate released from adipocytes increases dramatically (Pérez de Heredia et al., 2010). Alternatively, accumulating evidence indicates that lipids is the major energy source provided by adipocytes to tumor cells. CAAs release exogenous free fatty acids (FFAs), which are taken up by cancer cells via surface molecule CD36. FFAs could yield large quantity of ATP by increasing the rate of fatty acid β-oxydation (FAO) (Yang et al., 2020), thus contributing to the tumor progression and therapy resistance. In ovarian cancer cells, adipocytes adjust tumor metabolism by upregulating the expression of CD36, which was observed to regulate cellular metabolism including extracellular acidification, intracellular cholesterol accumulation and oxygen consumption, leading to substantial lipid droplets (LDs) accumulation and intracellular reactive oxygen species (ROS) increase (Ladanyi et al., 2018). Growing evidence has indicated that LDs function as organelles influencing chemotherapy response by storing excessive cholesterol and lipids (Guillaumond et al., 2015). Apart from CD36, FATPs (fatty acid transport protein family) and FABPpm (plasma membrane fatty acid binding protein) are reported to facilitate the FA transport process (Zhang M.M. et al., 2018). Likewise, adipocytes induce FABP4 expression, promoting metastasis and mediates carboplatin resistance in ovarian cancer cells. Meanwhile, knockdown of FABP4 leads to increased levels of DNA demethylation, downregulating genetic markers associated with OvCa metastasis and reducing the survival of clonal cancer cell, which provides theoretical support for targeting FABP combined with platinum treatment (Mukherjee et al., 2020).

### Secreting Various Factors

It is well acknowledged that aside from the energy storing function, adipose tissue is also a major endocrine organ producing a range of growth factors, adipokines, and adipocytokines, several of which have been demonstrated to confer therapy resistance (Duong et al., 2017).

The generation and secretion of leptin are found to be elevated in CAAs, compared to mature adipocytes. Previous studies have shown the up-regulated leptin could reduce toxicity of chemotherapy agents *in vitro* through the suppression of intracellular ROS. Further study revealed that adipocytes-derived leptin promotes myeloma cells proliferation and reduces the chemotherapy induced-apoptosis by mediating proliferation and apoptosis related proteins, which is related to the activation of JAK/STAT-PI3K/AKT pathway (Yu et al., 2016). Leptin could bind to the leptin receptor (OB-R), and then contributes to the drug resistance in melanoma cells via activating the PI3K/AKT and MEK/ERK signaling. Interestingly, siRNA knockdown of OB-Rb reverses the activation of AKT and ERK and enhances the chemosensitivity melanoma cells (Chi et al., 2014). Similar finding was reported in colon cancer cells, the combination of leptin and OB-R activates the PI3K/AKT signaling pathway, enhances tumor growth and promotes sphere formation through the overexpression of E-cadherin. Meanwhile, leptin could interfere with the efficacy of the chemotherapeutic agent 5-FU through promoting cell proliferation and suppressing 5-FU-induced apoptosis (Bartucci et al., 2010). In breast cancer cells, leptin participates in resistance to hormonal therapy. Leptin activates the estrogen receptor-alpha (ER-α) through the MAPK pathway regardless of the presence of estradiol, leading to nuclear localization of ER via promoting the expression of pS2, a classic estrogen-dependent gene. Consequently, the estrogen withdrawal status induced by aromatase inhibitors is alleviated, thus contributing to the progression of estrogen-dependent breast cancer (Catalano et al., 2004).

Adipocyte-secreted cytokines have been found to induce resistance via the modulation of cell death. IL-6, IL-8, MCP-1 are the most abundantly adipocyte-secreted cytokines (Duong et al., 2017). Recent studies suggested that increased expression and secretion of IL-6 in CAAs plays a key role in mediating breast cancer progression (Kim et al., 2018). In HER2-positive breast cancers, IL-6 expression can induce CSC-positive phenotype through activating NF-κB and STAT3 pathways, thus driving tumorigenesis and promoting chemotherapy resistance (Liu S. et al., 2018). SDF-1α, another adipocyte-derived chemoattractant, was reported to promote the migration of ALL toward adipose tissue, where leukemia cells could be protected from chemotherapy by remaining in a dormant state, thereby gaining survival advantage (Pramanik et al., 2013).

L-asparaginase (ASNase) is a major treatment regimen for ALL by depleting plasma asparagine and glutamine, while glutamine synthetase was found to be remarkedly elevated in CAAs in ALL. That means adipocytes prevent leukemic cell from ASNase induced cytotoxicity through directly increasing secretion of glutamine (Ehsanipour et al., 2013). Arachidonic acid (AA) is another chemo-protective lipid mediator secreted by adipocytes. It has been shown that AA significantly promotes the resistance of ovarian cancer cells to chemotherapeutic agents, partly dependent on AKT pathway. In addition, AA may participate in the modulation of drug resistance-related cell surface proteins including MRP1 and P-gP, influencing their organization, distribution and activity (Yang et al., 2019). Moreover, adipose-derived factors were demonstrated to confer the inhibition of trastuzumab-mediated ADCC in HER2^+^ breast tumor cells through decreasing the secretion of INF-γ in natural killer cells without altering their cytotoxicity. Further investigation remains to be done to determine the exact factor (Duong et al., 2015). Additional evidence suggests that adipocyte-derived adipokines activate JAK/STAT3 signaling and induce the upregulation of autophagic proteins, such as Atg5, Atg3, and LC3-I/II, activating autophagy thereby decreasing chemotherapy-induced apoptosis in myeloma cells, which highlights a novel therapeutic target for enhancing chemotherapy sensitivity in myeloma patients (Liu Z.Q. et al., 2015).

In addition to these factors, exosomes derived from CAAs also contribute to drug resistance in tumor cells, as a novel method of transmitting information among cells. Yeung et al. confirmed that CAAs-derived exosomes transfer higher levels of microRNA-21 (miR21) to ovarian cancer cells, which binds to a direct downstream target APAF1, known as a chemoresistance-associated gene, downregulating its expression, leading to paclitaxel resistance. Briefly, adipocytes derived exosomes could promote drug resistance and a malignant phenotype by decreasing the expression of APAF1 in ovarian cancer cells (Yeung et al., 2016).

### Altering Chemotherapy Pharmacokinetics

The concentration of active drugs is of particular importance in tumor treatment. However, it was found to be reduced in adipocyte-rich microenvironments, including adipose tissue, omentum, and bone marrow, leading to a localized decrease in the cytotoxic activity of chemotherapy, which partly accounts for chemoresistance (Sheng et al., 2017). Adipocytes alter chemotherapeutic pharmacokinetics mainly through two pathways, including increasing drug clearance and altering drug distribution. Anthracyclines are widely used in a variety of cancers as classical DNA-damaging agents, while adipocytes were demonstrated to highly expressed daunorubicin-metabolizing enzymes, aldo-keto reductases (AKR) and carbonyl reductases (CBR) isoenzymes to deactivate anthracyclines. Specifically, adipocytes reduce the antileukemia effect of daunorubicin by converting it to a less toxic metabolite, thereby reducing active drug concentration in the local microenvironment. Furthermore, Lehuédé et al. (2019) shown the doxorubicin subcellular distribution modified by adipocytes is associated with upregulating major vault protein (MVP). MVP is known as a major component of vault particles, involving in intracellular molecules transportation, including anti-cancer drugs. Therefore, increased expression of MVP in tumor cells induced by adipocytes increases drug efflux and decreases drug intracellular accumulation. Also, it has been demonstrated that overexpression of MVP clinically relates to drug resistance in human breast tumors, indicating a poor prognosis for chemotherapy (Lehuédé et al., 2019).

### Extracellular Matrix Remodeling

As previously mentioned, the dynamic adaptation of ECM is essential for cancer progression and invasion as well as drug resistance. Interestingly, adipocytes are vital source of ECM components. In breast tumors, adipocytes increased expression of matrix metalloproteinase-11 (MMP11), along with MMP1, MMP7, MMP10, and MMP14, involving in cisplatin resistance (Satoh et al., 1994; Choi et al., 2018). Collagen VI, another adipocyte-secreted ECM protein was reported to trigger tumor progression *in vivo* via activation of the NG2 receptor in cancer cells and sequentially inducing Akt, β-catenin, and cyclin D1 (Iyengar et al., 2005). Remarkably, the presence of collagen VI protein was found to promote resistance in cisplatin-sensitive ovarian cells, suggesting that reorganization of the EMC near the tumor is of great importance in drug resistance (Sherman-Baust et al., 2003). Furthermore, a cleavage product of collagen VI, endotrophin highly secreted from adipocytes upon chemotherapy was demonstrated to act as a signaling molecule enhancing EMT process, causing cisplatin resistance. Thiazolidinediones and the neutralization of endotrophin were found to increase chemo-sensitivity by suppressing EMT (Park et al., 2013).

Taken together, the aforementioned studies suggest the complicated role of adipocytes and lipid metabolism in the regulation of anticancer drug sensitivity (Table 6), which makes CAAs targeted therapy a potentially effective treatment. CAAs secrete metabolic substrates, adipokines, and cytokines and promote drug resistance, which may become a potential target for new treatments. For instance, targeting serum IL-6 by tocilizumab, an anti–IL-6 receptor antibody was demonstrated to improve prognosis and alleviate cachexia in chemotherapy-resistant patients with metastatic lung cancer (Ando et al., 2014). In addition, inhibition of CAAs lipolysis provides an opportunity of anticancer therapy. Mechanistically, inhibit the release of FFA and other metabolites from CAAs, thereby constrain the supply of lipid fuel to cancer cells. Alternatively, interference with FFA uptake provide another way to limit FFA supply to tumor cells. Usage of the CD36-neutralizing antibodies to inhibit fatty acid receptor CD36 leads to therapeutically suppression of tumor growth and metastasis in oral squamous cell carcinomas (Pascual et al., 2017). In ovarian cancer, small molecule inhibition of FABP4 diminishes fatty acids transfer and lipid accumulation in cancer cells, impeding intraabdominal metastasis and growth (Nieman et al., 2011). Recent study also demonstrated that BMS309403, a small molecule inhibitor of FABP4, markedly blocks cell proliferation as well as efficiently restores sensitivity of tumor cells to platinum both *in vitro* and *in vivo* (Mukherjee et al., 2020). However, it is still challenging to selectively target CAAs without affecting other cells. Therefore, identifying the specific molecular mechanisms of adipocyte-mediated drug resistance can help explore additional targets and develop new ways to combat drug resistance in combination with chemotherapy.

**TABLE 6:** Mechanisms of antitumor drug resistance mediated by CAAs.

Tumor type	Drug resistance	Mechanisms	References
ALL	ASNase	CAAs producing both ASN and GLN, counteracting the effects of ASNase.	Ehsanipour et al., 2013
	Vincristine	CAAs secreting SDF-1α, promoting the migration of ALL toward adipose tissue.	Pramanik et al., 2013
	Daunorubicin	CAAs decreasing daunorubicin concentration in ALL cells by expressing daunorubicin-metabolizing enzymes.	Sheng et al., 2017
Breast cancer	Trastuzumab	Adipose-derived factors decreasing the secretion of INF-γ in natural killer cells.	Duong et al., 2015
	Doxorubicin	CAAs inducing the overexpression of MVP in tumor cells, increasing drug efflux and decreasing drug intracellular accumulation.	Lehuédé et al., 2019
CRC	5-FU	CAAs secreting leptin activating the PI3K/AKT signaling pathway.	Bartucci et al., 2010
Myeloma	Bortezomib	CAAs derived leptin promoting the activation of JAK/STAT-PI3K/AKT pathway.	Yu et al., 2016
	Melphalan, bortezomib	CAAs derived adipokines activating JAK/STAT3 signaling and inducing the upregulation of autophagic proteins, promoting autophagy.	Liu Z. et al., 2015
Ovarian cancer	Paclitaxel	CAAs-derived exosomes transferring higher levels of miR21 to the cancer cells.	Yeung et al., 2016
	Cisplatin	CAAs releasing AA, activating the Akt pathway in ovarian cancer cells.	Yang et al., 2019
	Carboplatin	Adipocytes inducing FABP4 expression.	Mukherjee et al., 2020

*CAAs, cancer associated adipocytes; ALL, acute lymphoblastic leukemia; ASNase, L-asparaginase; ASN, amino acids asparagine; GLN, glutamine; ADCC, antibody-dependent cellular cytotoxicity; CRC, Colorectal cancer; 5-FU, 5-fluorouracil; miR21, microRNA-21; LDs, lipid droplets; ROS, reactive oxygen species; AA, Arachidonic acid; PDAC, pancreatic ductal adenocarcinoma.*

## Endothelial Cells

In addition to the cells mentioned above, endothelial cells (ECs), covering the inner surfaces of tumor blood vessels, are yet another vital stroma cells in tumor microenvironment that contribute to drug resistance (Hida et al., 2016). As a key mediator in blood vessel growth, ECs are now regarded as the main target cells for anti-vascular therapy. Cumulative evidence indicates that there is considerable heterogeneity in ECs. Tumor endothelial cells (TECs), a special subpopulation of ECs, are phenotypically and functionally different from normal endothelial cells (NECs), which exhibit the property of being quiescent and proliferate only once every 150 days (Hida et al., 2018). And it is the phenotypic and genotypic heterogeneities of TECs that offer novel mechanisms of resistance (Annan et al., 2020).

Cytogenetic abnormalities, including chromosomal aberration like aneuploidy and abnormal centrosomes, have been observed in TECs isolated from human and murine renal cell carcinoma (Akino et al., 2009; Maishi et al., 2019), suggesting that genetic instability is likely to account for the high frequency of resistance to chemotherapeutic drugs in TECs. TECs isolated from HCC were observed to possess enhanced angiogenic activity and acquire resistance to doxorubicin and 5-fluorouracil (Xiong et al., 2009). *In vivo*, several studies have elucidated that the expression of multidrug resistance protein 1 gene (MDR1) and its product, p-glycoprotein were increased in TECs compared to NECs, resulting in tumor growth and drug resistance (Huang et al., 2013; Kikuchi et al., 2020). MDR1 (ABCB1), MRP1 (ABCC1), and ABCG2 are all important ABC (ATP-binding cassette) transporters, which are known for conferring multidrug resistance by pumping drugs out of cells, decreasing intracellular drug concentrations (Gottesman et al., 2002). The up-regulation of MDR1 in TECs may be related to AKT activation caused by high level of VEGF secreted from tumor cells, which could be sufficiently impeded by the VEGFR kinase inhibitor, Ki8751 (Akiyama et al., 2012). Chemotherapy induced high IL-8 expression in tumor cells contributes to the upregulation of MDR1 expression in TECs, which in turn causes drug resistance in human bladder cancer cells (Kikuchi et al., 2020). Moreover, some studies showed that TECs express p-glycoprotein and confer resistance to anti-angiogenic drugs (Naito et al., 2016). Accordingly, drugs of P-glycoprotein inhibitor, such as verapamil, suppressed tumor angiogenesis and abrogated TEC resistance, and increased the sensitivity of tumor endothelium to chemotherapeutics (Akiyama et al., 2015; Bani et al., 2017). In a word, the high expression of P-glycoprotein and other ABC transporters in TECs plays a vital role in mediating multidrug resistance (Figure 3).

Hyperglycolytic metabolism has been noted as another major character of TECs versus NECs. The RNA sequencing of TECs and NECs revealed that the expression of most glycolytic genes are up-regulated in TECs, such as PFKFB3, encoding glucose transporter GLUT1 (Slc2a1). And this altered glycolytic metabolism is involved in the formation of abnormal tumor vessels, thus rendering chemoresistance by impairing the delivery and efficacy of chemotherapy. Targeting PFKFB3 in ECs, rather than angiogenic signals, to modulate ECs’ metabolism is an effective way to promote tumor vessel normalization (TVN), with increased chemotherapy sensitivity (Cantelmo et al., 2016).

The stemness properties of TECs was claimed to be responsible for resistance to anticancer drugs as well. It has been elucidated that TECs express stem cell markers including the ABC transporter, MDR1, as demonstrated above (Annan et al., 2020). Besides, aldehyde metabolism associated enzyme, namely aldehyde dehydrogenase (ALDH), is considered to be a stem cell marker in TECs. Hida et al. (2017) demonstrated that the increased ALDH^high^ TECs population induced by tumor-secreting factors in TME were accompanied by upregulation of stem-related genes such as MDR1, CD90, ALP, and Oct-4. That may be the cause of resistance to drug therapy.

Tavora et al. (2014) described that TEC-derived focal adhesion kinase (FAK) contributes to the acquisition of chemoresistance, via regulating DNA-damaging related NF-κB pathway and enhancing the subsequent production of cytokines from endothelial cells *in vivo* and vitro. Additionally, endothelial-cell FAK deficiency is sufficient to restore the sensitivity of tumor cells to DNA-damaging therapies through decreasing the activation of NF-κB and the following cytokine production. In consistence, the work is supported by clinical relevance as well. In lymphoma patients, disease progression is found to be correlated with altered endothelial cell FAK expression (Tavora et al., 2014).

Endothelial cells-derived paracrine factors, angiocrine factors, are highlighted in conferring drug resistance by the network of endothelial-carcinoma signaling interactions. Tumor-necrosis factor-α (TNF-α), an essential upstream activator of the NF-κB signaling cascade, was found to be strikingly upregulated in adjacent endothelial cells in breast cancer after doxorubicin chemotherapy treatment (Acharyya et al., 2012), which was induced by the overexpression of CXCL1/2 in breast cancer cells and S100A8/9 in CD11b^+^Gr1^+^ myeloid cells. Moreover, NF-κB activation amplified the CXCL1/2-S100A8/9 loop in turn. As a result, the highly activation of TNF-α-CXCL1/2-S100A8/9 paracrine network drives chemoresistance by activating ERK1/2, p38 MAPK, and p70S6K, mediating the pro-survival effect in tumor cells. Fibroblast growth factor (FGF) 4, originated from B cell lymphoma cells (LCs), was reported to activate FGFR1, which upregulated the expression of Notch ligand Jagged1 (Jag1) on neighboring ECs. Reciprocally, the elevated EC-derived angiocrine Jag1 promotes LC chemoresistance by activating Notch2–Hey1 signals in LCs. In brief, these results suggested the importance of FGF4-FGFR1/Jag1-Notch2 loop between LCs and ECs in fostering chemoresistance. Obviously angiocrine Jag1 is central to this loop and inhibiting the FGF4/Jag1 feed-forward signaling loop between LCs and ECs could sensitize LCs to chemotherapeutics such as doxorubicin (Cao et al., 2014). Furthermore, the importance of Notch signaling in drug resistance has been proven in other tumors. Hoarau-Vechot et al. (2019) demonstrated that AKT-activated ECs increase the expression of Jagged 1, activating notch signaling and inducing chemoresistance in ovarian cancer. Clinically, high Notch3 expression is notably associated with poorer overall survival and enhanced drug resistance. Thus, targeting of aberrant NOTCH signaling plays a critical role in suppressing ECs-mediated pro-tumoral niche in combination with other therapeutic strategies. IL-6, a proinflammatory cytokine increasingly secreted by other stroma cells, is an important angiocrine factor secreted by TECs as well. Bent et al. (2016) showed that strong expression of IL-6 from ECs was mediated by ROS-induced p38 activation *in vitro* under doxorubicin treatment. In consequence, IL-6 represses senescence-associated inflammation, promoting chemoresistance via the suppression of PI3K/AKT/mTOR pathway in B-cell lymphoma. Additionally, insulin growth factor (IGF) binding protein-7 (IGFBP7/angiomodulin), an important tumor-suppressive checkpoint expressed by TEC, was reported to participate in the TEC angiocrine mediated chemoresistance as well. Tumor derived FGF4 activates FGFR1 in endothelial cells and stimulates EST-2-dependent upregulation of IGF1 and suppression of IGFBP7, leading to chemotherapy tolerance and promoting tumor recurrence and lethality (Cao et al., 2017).

Moreover, it has been shown that ECs play an important part in mediating resistance to anti-vascular drugs and immunotherapy (Nagl et al., 2020). In glioblastoma, tumor derived PDGF activates ECs, inducing NF-κB-dependent Snail expression, resulting in endothelial-mesenchymal transformation (Endo-MT). Further, Endo-MT abrogates the expression of VEGFR-2, the major regulator of angiogenesis, causing endothelial resistance to anti-VEGF treatment (Liu T. et al., 2018). Remarkably, PDGFR-β knockout in ECs robustly sensitizes GBM tumors to anti-VEGF therapy, suggesting targeting PDGF/NF-κB/Snail axis and preventing Endo-MT in ECs may offer exciting opportunities in antiangiogenic therapy resistance. In addition to encasing blood vessels, endothelial cells also form the inner layer of lymphatic vessels, which implicates ECs also involve in treatment response to checkpoint antibody therapy as key mediators of immune regulation (Nagl et al., 2020). By detecting 1,592 endothelial cells in lung cancer, Lambrechts et al. (2018) revealed that TECs downregulate immune attraction pathways by reducing the expression of chemotaxis (CCL2, CCL18, and IL6), adhesion molecules (ICAM1) and antigen presentation associated molecular (major histocompatibility complex class I and II), which contributes to tumor immunotolerance in TME. Besides, TECs promote the formation of abnormal tumor vessels, which facilitates the presence of immunosuppressive immune cells, attenuating the efficacy of immunotherapy based on immune checkpoint inhibitors (ICIs) (Schaaf et al., 2018).

As discussed above, ECs induce resistance to chemotherapy, anti-vascular therapy, and immunotherapy through multiple mechanisms, including cytogenetic abnormalities, hyperglycolytic metabolism, secreting paracrine factors and inducing abnormal tumor vasculature (Table 7). Hence, targeting these crosstalks and signaling axis as stated before can help overcome tolerogenic events. In particular, reprogramming the ECs glycolytic phenotype and promoting tumor vasculature-normalizing, combined with immunotherapy are promising strategy (Stapor et al., 2014; Cantelmo et al., 2016).

**TABLE 7:** Mechanisms of antitumor drug resistance mediated by ECs.

Tumor type	Drug resistance	Mechanisms	References
B cell lymphoma	Doxorubicin	ECs producing Jag1, inducing Notch2-Hey1 in LCs.	Cao et al., 2014
	Doxorubicin	TECs releasing IL-6, promoting suppression of PI3K/AKT/mTOR pathway in B-cell lymphoma.	Bent et al., 2016
Breast cancer	Doxorubicin, cyclophosphamide	ECs secreting TNF-α, activating NF-κB pathway.	Acharyya et al., 2012
Glioblastoma	Anti-angiogenic drug	Hypoxia inducing the differentiation of tumor cells to ECs.	Soda et al., 2011
	Anti-angiogenic drug	Activated by PDGF, ECs inducing NF-κB-dependent Snail expression, resulting in VEGFR-2 down-expression and Endo-MT.	Liu T. et al., 2018
HCC	Doxorubicin	Abnormal activation of FGFR1-ETS2 pathway in TECs	Cao et al., 2017
	Anti-angiogenic drug	CD105^+^ TECs showing increased apoptosis resistance and motility and proangiogenic properties.	Xiong et al., 2009
Lung cancer	Anti-angiogenic drug	ECs contributing to new blood vessel formation.	Naito et al., 2016
Melanoma	Doxorubicin	TEC-derived FAK regulating DNA-damaging related NF-κB pathway.	Tavora et al., 2014
Ovarian cancer	Paclitaxel, doxorubicin, vincristine	Highly expression of P-gp and other ABC transporters in TECs.	Bani et al., 2017
RCC	Fluorouracil	TEC containing stem cell-like populations with high ALDH activity ALDH^high^ TEC	Hida et al., 2017
Urothelial carcinoma	Paclitaxel	Increased tumor IL-8 secretion promoting TECs express high levels of a drug efflux transporter, ABCB1.	Kikuchi et al., 2020

*ECs, endothelial cells; TECs, tumor endothelial cells; LCs, lymphoma cells; TNF-α, tumor-necrosis factor-α; PDGF, platelet-derived growth factor; Endo-MT, endothelial-mesenchymal transformation; HCC, hepatocellular carcinoma; FAK, focal adhesion kinase; P-gp, P-glycoprotein; ABC transporters, ATP-binding cassette transporters; RCC, renal cell carcinoma; 5-FU, 5-fluorouracil; ALDH, aldehyde dehydrogenase.*

## Platelets

Derived from common hematopoietic progenitor cells, platelet is a key regulator in hemostasis and thrombosis (Contursi et al., 2017). However, emerging studies have focused on its contributions to various aspects of cancer progression, including angiogenesis, tumor growth, metastasis and drug resistance (D’Alessandro et al., 2015; Tesfamariam, 2016; Contursi et al., 2017). Multiple reports have shown a strong link between platelets and the efficiency of tumor chemotherapy. For instance, platelet-sparing phenomenon was reported in patients treated with carboplatin and paclitaxel chemotherapy (Pertusini et al., 2001). Moreover, differential platelet levels or the platelet/lymphocyte ratios have been suggested to be a potential predictive factor of chemotherapy response or survival prognosis in various tumors, including breast cancer, gastric cancer, NSCLC, pancreatic neuroendocrine tumor, ovarian cancer and so on (Bottsford-Miller et al., 2015; Cuello-López et al., 2018; Fang et al., 2018; Gong et al., 2018; Xu S.S. et al., 2019). Tumor-educated platelets (TEPs) are even considered to be potential non-invasive biomarker in achieving effective cancer management (In ’t Veld and Wurdinger, 2019). Clinically, high platelet count has been correlated with chemotherapy resistance. Therefore, it is necessary to understand the association between platelets and drug-resistance in TME.

Platelets contribute to drug resistance through secreting various factors, altering chemotherapy pharmacokinetics, and eliciting multiple signaling pathways (Table 8).

**TABLE 8:** Mechanisms of antitumor drug resistance mediated by platelets.

Tumor type	Drug resistance	Mechanisms	References
Adenocarcinoma	Paclitaxel	Platelets secreting thrombospondin-1, and RANTES, modulating cancer cell cycle, DNA damage repair pathways and MAPK levels.	Radziwon-Balicka et al., 2012
HCC	Multikinase inhibitor	Platelets derived growth factors such as PDGF, TGF-α and -β, EGF and serotonin, regulating the sensitivity and resistance to multikinase inhibitors.	D’Alessandro et al., 2015
NSCLC	Cisplatin	Platelets rescuing cisplatin-induced apoptosis via the Akt/Bad/Bcl-2 signaling pathway.	Wang Z. et al., 2018
Pancreatic cancer	Cisplatin	Platelets releasing TGF-β, activating PI3K/Akt and MEK/Erk signaling.	Chen et al., 2013

*HCC, hepatocellular carcinoma; PDGF, platelet-derived growth factor; EGF, epidermal growth factor; NSCLC, non-small cell lung cancer.*

Many reports have indicated platelets to be an important source of diverse growth factors and cytokines, leading to drug resistance in TME (Assoian et al., 1983). As mentioned earlier, TGF-β/NF-κB signaling is a vital pathway involved in tumor drug resistance. Recent experiment demonstrated that platelets not only function as the major source of TGF-β but also express the cell surface-docking receptor glycoprotein A repetitions predominant (GARP), increasing the active TGF-β levels. The GARP-TGF-β axis promoted by platelets constrain T cell immunity against cancer, thus rendering resistance to therapy (Rachidi et al., 2017). Besides, platelet-derived TGF-β could activate the TGF-β/Smad and NF-κB pathways, subsequently leading to the EMT in tumor cells (Labelle et al., 2011), which was suggested to significantly contribute to the development of chemoresistance (Fischer et al., 2015). In pancreatic cancer, TGF-β released by platelets diminished cisplatin sensitivity through activating PI3K/Akt and MEK/Erk signaling (Chen et al., 2013). Apart from TGF-β, other platelet factors were reported to mediate drug resistance *in vitro* as well. Clusterin, thrombospondin-1, and RANTES secreted by platelet are demonstrated to protect adenocarcinoma cells from anticancer drugs via modulating cancer cell cycle, DNA damage repair pathways and MAPK levels (Radziwon-Balicka et al., 2012).

Another important mechanism lies in modulating drug metabolism. Clinical studies have shown the association of multidrug resistance-associated protein 1 (MRP1) expression and platelet count with clinical outcome in 427 operable NSCLC patients (Wang Z. et al., 2018). In fact, the expression of two ABC transporter in platelets, MRP4 and MRP1, was suggested to be relatively high (Köck et al., 2007), which confers drug resistance by promoting drug efflux, reducing intracellular drug concentrations (Oevermann et al., 2009).

In addition to chemotherapy drugs, platelets can also lead to tolerance to anti-angiogenic therapy. In HCC, platelets derived growth factors such as PDGF, TGF-α, and -β, EGF and serotonin, are ascribed to modulate the sensitivity and resistance to multikinase inhibitors (D’Alessandro et al., 2015). In glioblastoma multiforme (GBM) patients, platelet-derived VEGF shows potent pro-angiogenic effects on GBM-derived endothelial cells. Patients with GBM have significantly higher intraplatelet VEGF concentrations compared to healthy controls (Di Vito et al., 2017). Consistently, it has been accepted that redundancy of vascular stimulation signals may be the basis for resistance to anti-angiogenic therapy (van Beijnum et al., 2015).

Accordingly, in terms of platelet-induced drug resistance, apart from targeting relevant platelet-originated factors (e.g., PDGF, TGFβ1, and EGF) and pathways including TGF-β/NF-κB signaling, targeting the activity of platelets in TME is an important approach to improve the sensitivity of tumor therapy. Aspirin, a potent inhibitor of platelet function, has been suggested to potentially enhance the sensitivity to 5-Fu-based chemotherapy in colorectal cancer (CRC) via abrogating the activation of NF-κB both *in vivo* and *in vitro* (Fu et al., 2019). Ticagrelor, an inhibitor of the ADP–P2Y12 axis, was demonstrated to boost chemotherapeutic efficacy synergized with chemotherapeutic agents in pancreatic cancer cells, which was mediated by reducing EGF-dependent AKT activation (Elaskalani et al., 2020). Noteworthily, potential challenges remain in antiplatelet strategies during cancer therapy, including the risk of bleeding and comorbidity. Further experimental and clinical confirmation validation are warranted to validate the benefits and risks of combined antiplatelet therapy in cancer patients.

## Conclusion

Taken together, the evidence we summarized here clearly demonstrate that TME has a considerable impact on tumorigenesis and resistance in the context of therapeutic intervention. Therefore, TME is an attractive target for both sensitizing tumors to traditional therapies and as an alternative choice for resistant disease. Targeting these stroma cells is a potentially helpful adjunct to immunotherapy and targeted therapy. Repolarization and normalization rather than elimination of these cells seems a preferable choice. Despite of the heat of immunotherapy, we cannot ignore the fact that chemotherapies and molecular targeted therapies are still basic and effective in many cancers, especially for early-stage patients. The primary challenges lie in that we need to determine the best combinations or sequences and manage patients based on whether they are likely to benefit from a specific drug or drug combination.

In any case, our current insight into the cancer therapy suggests that rapid tumor cell elimination is crucial to prevent acquired therapeutic resistance. In order to achieve this goal, treatment combinations need to be optimized to delay the progression of drug resistance. Meanwhile, novel strategies targeting and reshaping the microenvironment to block pro-tumor cross-talk and restore immune surveillance are urgently required. Lastly, because current preclinical models of TME cannot completely represent variations exhibited by patients of different histological types, detailed molecular and cellular profiles are needed for clinical trials, which is necessary for identifying predictive biomarkers and promoting the development of personalized treatment.

## Author Contributions

YN and XTZ contributed to the first draft of the article, the tables, and the figures. JY provided assistance in making figures. HS and HL revised the manuscript. XZ and XM conceived the presented idea, revised the manuscript again, and approved the final version. All authors approved the manuscript for publication.

## Conflict of Interest

The authors declare that the research was conducted in the absence of any commercial or financial relationships that could be construed as a potential conflict of interest.
